# Strong and lasting impacts of past global warming on baleen whales and their prey

**DOI:** 10.1111/gcb.16085

**Published:** 2022-02-02

**Authors:** Andrea A. Cabrera, Elena Schall, Martine Bérubé, Pia Anderwald, Lutz Bachmann, Simon Berrow, Peter B. Best, Phillip J. Clapham, Haydée A. Cunha, Luciano Dalla Rosa, Carolina Dias, Kenneth P. Findlay, Tore Haug, Mads Peter Heide‐Jørgensen, A. Rus Hoelzel, Kit M. Kovacs, Scott Landry, Finn Larsen, Xênia M. Lopes, Christian Lydersen, David K. Mattila, Tom Oosting, Richard M. Pace, Chiara Papetti, Angeliki Paspati, Luis A. Pastene, Rui Prieto, Christian Ramp, Jooke Robbins, Richard Sears, Eduardo R. Secchi, Mónica A. Silva, Malene Simon, Gísli Víkingsson, Øystein Wiig, Nils Øien, Per J. Palsbøll

**Affiliations:** ^1^ Groningen Institute for Evolutionary Life Sciences University of Groningen Groningen The Netherlands; ^2^ GLOBE Institute University of Copenhagen Copenhagen Denmark; ^3^ Center for Coastal Studies Provincetown Massachusetts USA; ^4^ Swiss National Park Chastè Planta‐Wildenberg Zernez Switzerland; ^5^ Natural History Museum University of Oslo Oslo Norway; ^6^ Marine and Freshwater Research Centre Galway‐Mayo Institute of Technology Galway Ireland; ^7^ Irish Whale and Dolphin Group Merchants Quay Kilrush County Clare Ireland; ^8^ Department of Zoology and Entomology Mammal Research Institute University of Pretoria Hatfield South Africa; ^9^ Seastar Scientific Inc. Vashon Island Washington USA; ^10^ Aquatic Mammals and Bioindicators Laboratory (MAQUA) Faculty of Oceanography State University of Rio de Janeiro ‐ UERJ Maracanã Rio de Janeiro Brazil; ^11^ Genetics Department of the Biology Institute State University of Rio de Janeiro ‐ UERJ Maracanã Rio de Janeiro Brazil; ^12^ Laboratory of Ecology and Conservation of Marine Megafauna Institute of Oceanography Federal University of Rio Grande‐FURG Rio Grande Rio Grande do Sul Brazil; ^13^ Department Conservation and Marine Sciences Centre for Sustainable Oceans Economy Cape Peninsula University of Technology Cape Town South Africa; ^14^ Research Group Marine Mammals Institute of Marine Research Tromsø Norway; ^15^ Greenland Institute of Natural Resources Nuuk Denmark; ^16^ Department of Biosciences Durham University Durham UK; ^17^ Norwegian Polar Institute Tromsø Norway; ^18^ Section for Ecosystem based Marine Management National Institute of Aquatic Resources Technical University of Denmark Kongens Lyngby Denmark; ^19^ School of Biological Sciences Victoria University of Wellington Wellington New Zealand; ^20^ Northeast Fisheries Science Center National Marine Fisheries Service Woods Hole Massachusetts USA; ^21^ Department of Biology University of Padova Padova Italy; ^22^ Hellenic Agricultural Organisation‐“DIMITRA” Herakleion Crete Greece; ^23^ Institute of Cetacean Research Tokyo Japan; ^24^ Institute of Marine Sciences – Okeanos & Institute of Marine Research ‐ IMAR University of the Azores Horta Portugal; ^25^ Sea Mammal Research Unit Scottish Oceans Institute University of St. Andrews Scotland UK; ^26^ Mingan Island Cetacean Study Saint Lambert Québec Canada; ^27^ Greenland Climate Research Centre Greenland Institute of Natural Resources Nuuk Greenland; ^28^ Marine and Freshwater Research Institute Hafnarfjörður Iceland; ^29^ Marine Mammal Division Institute of Marine Research Bergen Norway

**Keywords:** cetaceans, climate change, demographic inference, genetics, glaciation, marine ecosystem, North Atlantic Ocean, polar ecosystems, Southern Ocean

## Abstract

Global warming is affecting the population dynamics and trophic interactions across a wide range of ecosystems and habitats. Translating these real‐time effects into their long‐term consequences remains a challenge. The rapid and extreme warming period that occurred after the Last Glacial Maximum (LGM) during the Pleistocene–Holocene transition (7–12 thousand years ago) provides an opportunity to gain insights into the long‐term responses of natural populations to periods with global warming. The effects of this post‐LGM warming period have been assessed in many terrestrial taxa, whereas insights into the impacts of rapid global warming on marine taxa remain limited, especially for megafauna. In order to understand how large‐scale climate fluctuations during the post‐LGM affected baleen whales and their prey, we conducted an extensive, large‐scale analysis of the long‐term effects of the post‐LGM warming on abundance and inter‐ocean connectivity in eight baleen whale and seven prey (fish and invertebrates) species across the Southern and the North Atlantic Ocean; two ocean basins that differ in key oceanographic features. The analysis was based upon 7032 mitochondrial DNA sequences as well as genome‐wide DNA sequence variation in 100 individuals. The estimated temporal changes in genetic diversity during the last 30,000 years indicated that most baleen whale populations underwent post‐LGM expansions in both ocean basins. The increase in baleen whale abundance during the Holocene was associated with simultaneous changes in their prey and climate. Highly correlated, synchronized and exponential increases in abundance in both baleen whales and their prey in the Southern Ocean were indicative of a dramatic increase in ocean productivity. In contrast, the demographic fluctuations observed in baleen whales and their prey in the North Atlantic Ocean were subtle, varying across taxa and time. Perhaps most important was the observation that the ocean‐wide expansions and decreases in abundance that were initiated by the post‐LGM global warming, continued for millennia after global temperatures stabilized, reflecting persistent, long‐lasting impacts of global warming on marine fauna.

## Graphical abstract

Genetic analyses revealed that past global warming had strong, ocean‐wide impacts on baleen whales and their prey in the North Atlantic and Southern Oceans. The Holocene was characterized by a global increase in baleen whale abundance associated with changes in prey and climate. The changes initiated by the rapid global warming 7–12 thousand years ago continued to affect abundance long after the global temperatures stabilized. These findings warn of the potential of dramatic long‐term effects from current climate change on the marine ecosystem.

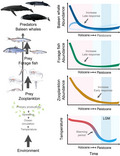

## 1 INTRODUCTION

Global warming is affecting species distributions, population dynamics, and trophic interactions across Earth (Parmesan, 2006; Post et al., 2009; Scheffers et al., 2016; Trucchi et al., 2014). However, predicting the long‐term demographic consequences of global warming at the species level, among groups of species and trophic webs remains challenging (Abrantes, 2000; Hasselmann et al., 2003). Understanding biological responses to past periods of global warming, such as the rapid and extreme warming period during the Pleistocene–Holocene transition 7–12 thousand years ago (kya) can potentially provide new insights into the trajectory of future biological change (Tornqvist & Hijma, 2012). Increases in global temperatures by an average of 15°C during the Pleistocene–Holocene transition led to large‐scale physical and environmental changes, which in turn led to extensive species redistributions and changes in abundance in a wide range of terrestrial taxa (Brüniche‐Olsen et al., 2021; Hewitt, 2000; Lorenzen et al., 2011; Lyons et al., 2010). Although the effects of the Pleistocene–Holocene transition on terrestrial ecosystems have been well documented, the responses of marine megafauna, such as baleen whales, to these changes remain poorly understood.

With a few exceptions, baleen whales are globally distributed megafauna that feed on invertebrates and fish. Most baleen whale populations undertake extensive, seasonal migrations between low‐latitude winter breeding grounds and high‐latitude summer feeding areas (Lockyer & Brown, 1981). The trophic position and ocean‐wide range of baleen whales suggests that they are subject to environmental and ecological changes across entire ocean basins. Consequently, it is reasonable to hypothesize that the ocean‐wide ecological changes caused by past periods of global warming are mirrored in the long‐term demographic history of baleen whales.

Analyses of intra‐specific genetic variation can be utilized to gain insights into the responses of species to past climate oscillations and how past climate oscillations structured contemporary ecosystems (for an overview see Colella et al., 2020). Herein, DNA sequences from more than 7000 specimens were employed to infer the demographic histories during the last 30 ky in eight baleen whale species and seven prey species (i.e., fish, krill, and copepods); a period when Earth and its oceans underwent substantial global warming. In order to disentangle the intertwined effects of baleen whale feeding ecology and oceanic context, this study assessed the long‐term demographic responses in both baleen whales and their prey across two ocean basins with contrasting oceanographic characteristics (Seibold et al., 2018); the Southern Ocean and the North Atlantic Ocean.

**FIGURE 1:**
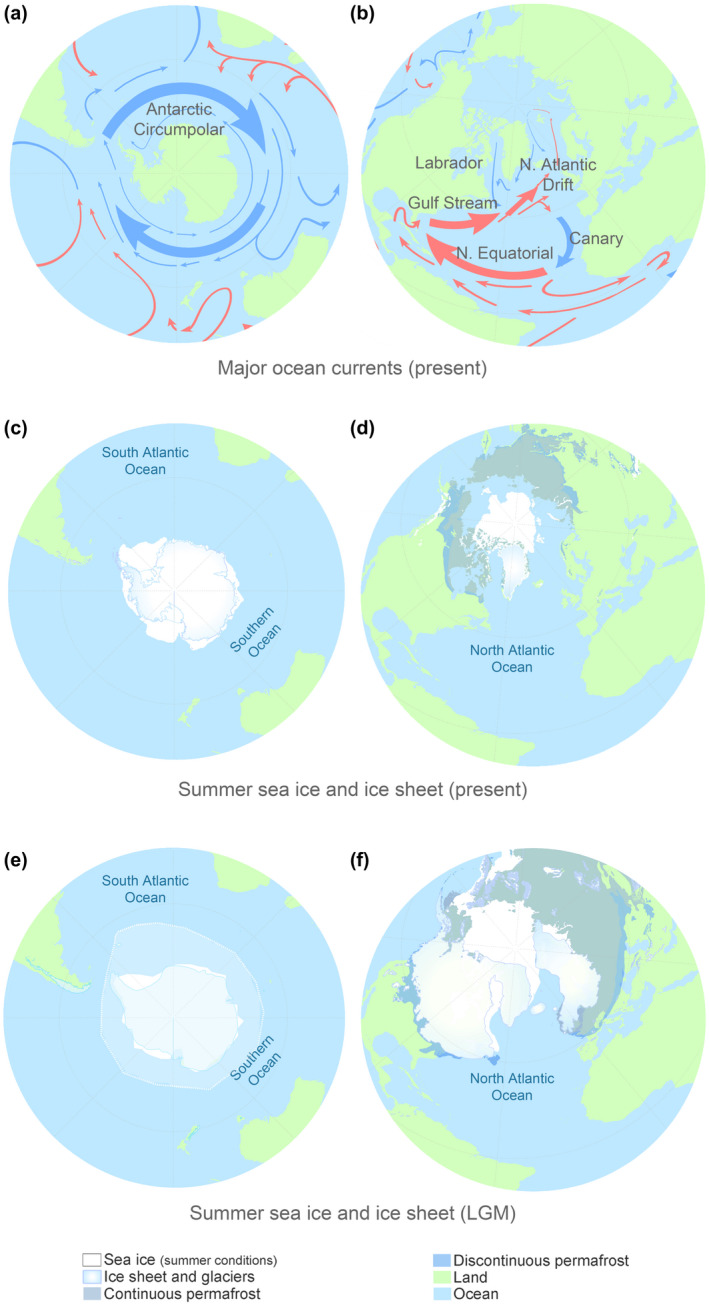
Major ocean currents and summer sea ice conditions before and after the Pleistocene‐Holocene transition. (a, b) Simplified depictions of the major surface ocean currents in the Southern and North Atlantic oceans. Blue and red lines indicate cool and warm currents, respectively. (c, d) Approximate contemporary summer ice coverage. (e, f) Inferred summer sea and land ice coverage during the LGM

The Southern and the North Atlantic Oceans differ in large‐scale oceanographic features (Figure 1). The Southern Ocean is a large ocean basin dominated by the wide and persistent Antarctic Circumpolar Current (Figure 1a; Tynan, 1998) and a comparatively stable pelagic food web that is largely centered around a single species, the Antarctic krill (*Euphausia superba*; Hopkins, 1985). In contrast, the much smaller North Atlantic Ocean is influenced by multiple, interacting, warm and cold ocean currents as well as continental run‐off and cyclic climate oscillations (Figure 1b; O'Hare et al., 2005; Rossby, 1996). The pelagic food web in the North Atlantic Ocean is more complex and dynamic in terms of baleen whale prey compared to the Southern Ocean (Kortsch et al., 2019). In the Southern Ocean, most baleen whale species feed primarily on krill, whereas the same baleen whale species have a more diverse diet in the North Atlantic Ocean (Bluhm & Gradinger, 2008; Kawamura, 1980; Víkingsson et al., 2014). The two oceans also differ in key physical features, such as the extent of seasonal sea ice, as well as the velocity of sea ice reduction during the global warming after the Last Glacial Maximum (LGM), 19–26 kya (Figure 1c–f; Clark et al., 2009; Spindler, 1990).

To the best of our knowledge, no previous study has assessed the correlation in the long‐term demographic responses to climate change across multiple predators and their prey on such large spatial scales.

## 2 MATERIALS AND METHODS

### 2.1 Taxon selection

**FIGURE 2:**
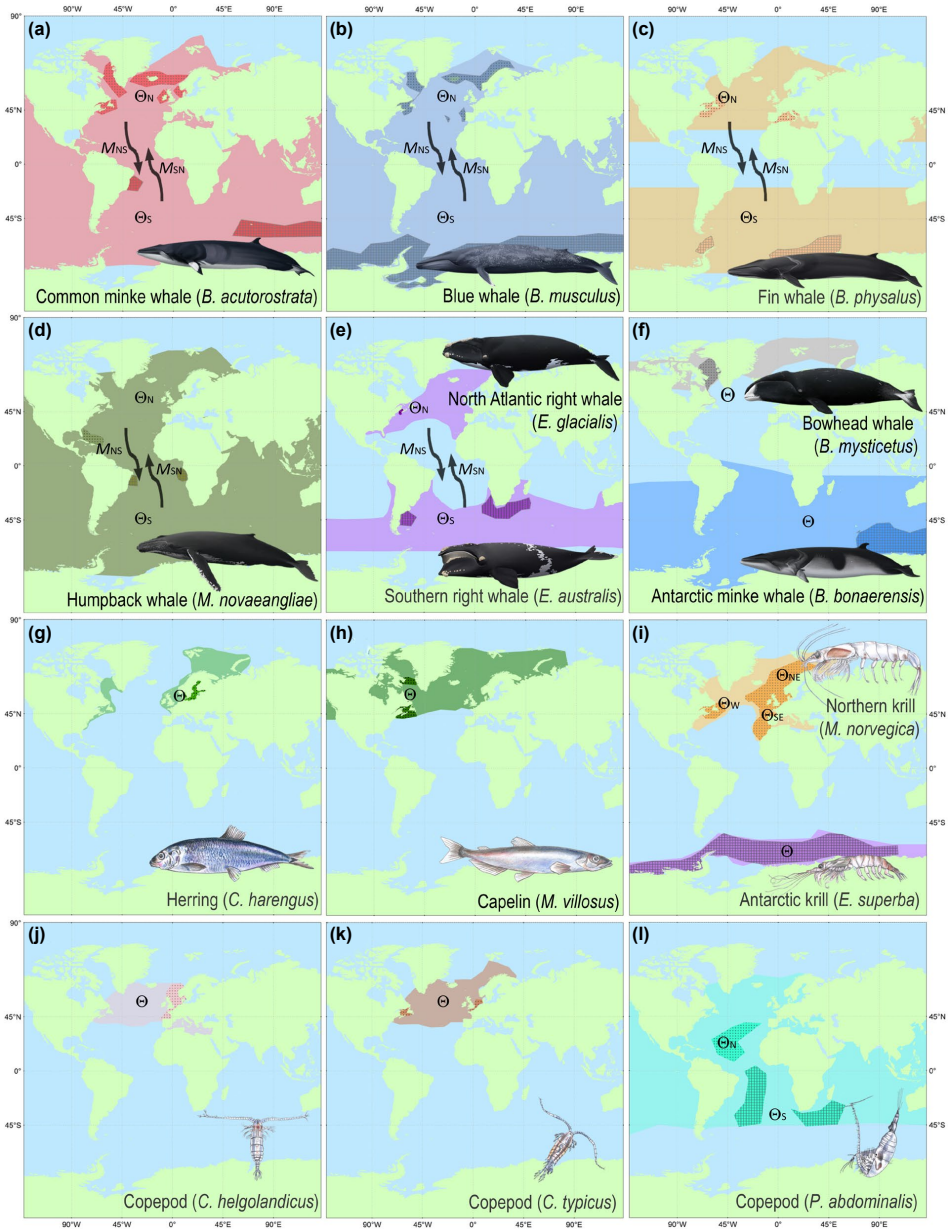
Species ranges, sampling location and estimated genetic parameters for baleen whales and their prey in the Southern and North Atlantic oceans. Color shaded areas represent approximated contemporary species ranges. Accentuated areas denote sampling locations. Θ (proxy for abundance) and *M* (gene flow) denote estimates in each species. N: northern, S: southern, NE: northeastern, W: western and SE: southeastern populations. Arrows represent the direction of *M* (*M*_NS_: from the North Atlantic Ocean into the Southern Ocean, *M*_SN_: from Southern into the North Atlantic Ocean)

This study focused on eight baleen whale species as well as seven fish and invertebrate species, representing baleen whale prey (Figure 2; Table 1). Four of the selected baleen whale species have global distributions: the common minke whale (*Balaenoptera acutorostrata*), the blue whale (*B*. *musculus*), the fin whale (*B*. *physalus*), and the humpback whale (*Megaptera novaeangliae*). By contrast, the distribution of the North Atlantic right whale (*Eubalaena glacialis*) is limited to the North Atlantic Ocean and the southern right whale (*E*. *australis*) to the Southern Hemisphere (Figure 2a–e). The bowhead whale (*Balaena mysticetus*) is mostly a High Arctic resident (Figure 2f) and the Antarctic minke whale (*B*. *bonaerensis*) is mainly confined to the Southern Ocean (Figure 2f).

**TABLE 1:** Species, sampling region, sample size, and genetic marker

Species	Common name	Sampling region	*n*	Marker	Sequence length/number of SNPs^a^	Source
Mitochondrial data						
North Atlantic Ocean						
Baleen whales						
*Balaenoptera acutorostrata*	Common minke whale	NA	931	CR	322	This study
*Balaenoptera musculus*	Blue whale	NA	325	CR	404	This study
*Balaenoptera physalus*	Fin whale	WNA	280	CR	391	This study, Archer et al. (2013)
*Megaptera novaeangliae*	Humpback whale	WI	1086	CR	396	This study
*Eubalaena glacialis*	North Atlantic right whale	WNA	269	CR	381	Malik et al. (1999)
*Balaena mysticetus*	Bowhead whale	WNA	395	CR	454	This study
Prey species						
*Meganyctiphanes norvegica*	Northern krill	NA	834^d^	DI	155	Papetti et al. (2005), Zane et al. (2000)
*Calanus helgolandicus*	Copepod^c^	ENA	218	16S	408	Yebra et al. (2011)
*Centropages typicus*	Copepod^b^	NA	79	COI	560	Castellani et al. (2012)
*Pleuromamma abdominalis*	Copepod^c^	NA	130	COI	441	Hirai et al. (2015)
*Clupea harengus*	Atlantic herring^b^	ENA	98	COI	1551	Teacher et al. (2012)
*Mallotus villosus*	Capelin^b^	WNA	41	CYTB	572	Colbeck et al. (2011)
Southern Ocean						
Baleen whales						
*Balaenoptera acutorostrata*	Common minke whale	WSA, SO	23	CR	322	Pastene et al. (2007)
*Balaenoptera musculus*	Blue whale	SO	230	CR	404	LeDuc et al. (2008), Sremba et al. (2012)
*Balaenoptera physalus*	Fin whale	SO	61	CR	391	This study, Archer et al. (2013)
*Megaptera novaeangliae*	Humpback whale	SA	500	CR	396	Jackson et al. (2014)
*Eubalaena australis*	Southern right whale	SA	481	CR	381	This study, Valenzuela et al. (2009)
*Balaenoptera bonaerensis*	Antarctic minke whale	WSA, SO	180	CR	337	Pastene et al. (2007)
Prey species						
*Euphausia superba*	Antarctic krill^b^	SO	640	COI	593	Goodall‐Copestake et al. (2010), Deagle et al. (2015)
*Pleuromamma abdominalis*	Copepod^c^	SA, WIO	231	COI	441	Hirai et al. (2015)
Nuclear data						
*B. acutorostrata*	Common minke whale	NA	27	SNPs	14,304 (×10) 24,988 (×2)	This study
*E. australis*	Southern right whale	SA	45	SNPs	31,482 (×10) 68,575 (×2)	This study
*B. physalus*	Fin whale	NA	28	SNPs	29,544 (×10) 56,325 (×2)	This study

Note

Abbreviations: *n*, number of samples. Sampling region: NA, North Atlantic; ENA, eastern NA; WNA, western NA; WI, West Indies; SA, South Atlantic; WSA, western SA; SO, Southern Ocean; WIO, western Indian Ocean. Marker: 16S, mitochondrial 16S; COI, mitochondrial cytochrome *c* oxidase, subunit I; CR, mitochondrial control region; CYTB, mitochondrial cytochrome *b*; ND1: mitochondrial NADH dehydrogenase, subunit I.

^a^ Sequence length in number of base pairs or number of estimated SNPs (i.e., number of inferred polymorphic sites from the site frequency spectrum) for each species with minimum coverage at ×10 and ×2.

^b^ Reported as whale prey species.

^c^ Occupies a similar ecological niche as known baleen whale prey.

^d^ Includes 654 sequences from the northeastern NA (NE‐NA), 146 from the southeastern NA (SE‐NA) and 34 from the western NA (W‐NA).

The fish and invertebrate species (Figure 2; Table 1) included two krill species, the Antarctic (*E*. *superba*) and the Northern krill (*Meganyctiphanes norvegica*), three copepods (*Centropages typicus*, *Calanus helgolandicus,* and *Pleuromamma abdominalis*), and two schooling fish species, the North Atlantic herring (*Clupea harengus*) and the capelin (*Mallotus villosus*). These species are known baleen whale prey or occupy a similar ecological niche as known baleen whale prey species thus were used as proxies for baleen whale prey (Table 1). Antarctic krill is found in the Southern Ocean, whereas the Northern krill, the copepods *Ce*. *typicus* and *Ca*. *helgolandicus*, as well as the fishes, are all Northern Hemisphere species. The copepod, *P*. *abdominalis*, has a wider distribution, including the Northern and Southern Hemisphere (Figure 2g–l).

### 2.2 Sample collection

The specimens were collected in the North and South Atlantic Oceans, the southwestern Indian Ocean, and the Southern Ocean. The latter three areas are collectively referred to as the Southern Ocean (Table 1).

Skin samples from free‐ranging baleen whales were collected using remote biopsy sampling techniques (Palsbøll et al., 1991). Additionally, some samples were obtained during necropsies from beached carcasses, fisheries bycatches as well as local subsistence or commercial whaling operations prior to the moratorium on commercial whaling in 1986. Tissue samples were preserved in 5 m NaCl with 20% dimethyl sulfoxide (Amos & Hoelzel, 1991) and stored at −20 or −80°C. Total‐cell DNA was extracted from tissue samples using either standard phenol and chloroform extraction processes (Sambrook et al., 1989) or DNeasy™ columns (Qiagen, Inc.) following the manufacturer's instructions.

Multi‐locus microsatellite genotypes (data not included) were employed to identify and remove duplicate samples collected from the same individual and to identify closely related individuals sampled in a non‐independent manner (e.g., mother and calf). Closely related individuals sampled at random, that is, during different sightings, were retained in the data set (Waples & Anderson, 2017).

### 2.3 Mitochondrial DNA sequence data

Mitochondrial DNA (mtDNA) sequence data were obtained from different regions of the mitochondrial genome (Table 1). These included; the control region (CR), cytochrome *c* oxidase subunit I (COI), NADH dehydrogenase subunit I (NDI), cytochrome *b* (CYTB), and 16S ribosomal DNA (16S rDNA). mtDNA sequence data were either generated during this study or obtained from previously published studies (Table 1). The vast majority of published baleen whale mtDNA sequences cover the 5′‐end of mitochondrial CR. Therefore, using these sequences facilitates access to large data sets, which comprise the most variable part of the mitochondrial genome. Accordingly, new baleen whale mtDNA sequences generated for this study targeted the same region in the manner described below. The Northern krill data set was generated using a single‐strand conformation polymorphism (SSCP) protocol to detect different DNA sequences (i.e., haplotypes). Only different electromorhps were sequenced implying that some sequence variation may have gone undetected.

#### 2.3.1 Laboratory analyses

The first ~400 bp of the 5′ end of the mitochondrial CR were amplified using the DNA oligo‐nucleotides MT4F (Árnason & Gullberg, 1993) and BP16071R (Drouot et al., 2004). The initial polymerase chain reaction (PCR; Mullis & Faloona, 1987) amplifications were performed in a 20 μl reaction volume with 0.2 μm of each dNTP, 67 mm Tris‐HCl (pH 8.8), 2 mm MgCl_2_, 17 mm NH_3_SO_4_, 10 mm β‐mercaptoethanol, 0.1 μm of each oligo‐nucleotide, 0.4 U of *Taq* DNA polymerase (Fermentas, Inc.) and ~10–20 ng of extracted DNA. The thermo‐cycling conditions were: 2′ (min) at 94°C, followed by 25 cycles each with 15′′ (s) at 94°C, 30′′ at 54°C, and 120′′ at 72°C. Unincorporated primers were degraded and excess nucleotides were removed using *shrimp alkaline phosphatase* and *exonuclease* I as described by Werle et al. (1994). Cycle sequencing was conducted according to the manufacturer's instructions (using 1/16th of the recommended amount of Big Dye™ v3.1 Terminator Ready Reaction Mix; Life Technologies, Inc.) with the oligo‐nucleotides MT4F or BP16071R. Excess nucleotides and oligo‐nucleotides were removed by ethanol precipitation and the cycle‐sequencing products were re‐suspended in 10 μl deionized formamide (Calbiochem, Inc.). The order of cycle‐sequencing products was resolved by capillary electrophoresis on Applied Biosystems ABI Prism™ 3730 (Life Technologies, Inc.). The resulting chromatograms were visually inspected using CHROMAS™ (ver. 2.13; Technelysium Pty Ltd.) and SEQUENCHER® (ver. 5.1; Gene Codes Corporation).

#### 2.3.2 Data processing and sequence alignment

Mitochondrial DNA sequences were aligned using the CLUSTALW algorithm (Thompson et al., 1994) as implemented in MEGA (ver. 6.0, Tamura et al., 2013) with default parameter settings. All DNA sequences of a particular locus and species were trimmed to the same length (Table 1).

#### 2.3.3 Diversity estimation

Descriptive genetic diversity indices were estimated as implemented in DNASP v.6.12.03 (Rozas et al., 2017) for each species and ocean basin. The indices were; the number of segregating sites (*S*), number of haplotypes (*h*), haplotype (*H*) and nucleotide (*π*) diversity (Nei, 1987), as well as Tajima's *D* (Tajima, 1989) and Fu's *F* statistic (Fu, 1997). Nucleotide sites subject to insertions or deletions and missing data were excluded from the analyses.

#### 2.3.4 Estimation of changes in genetic diversity and migration rates from single‐locus DNA sequences

Temporal changes in regional genetic diversity (Θ) and immigration rates (*M)* were employed as proxies for inferences of changes in abundance and connectivity, respectively. The genetic diversity in a population is determined by the composite parameter Θ, the product of the mutation rate, and the effective population size (Watterson, 1975), which in turn, is a function of the census population size or abundance. *M* denotes the probability (scaled with the mutation rate) per generation that an individual is an immigrant. The temporal changes in Θ and *M* were estimated using the approach implemented in MIGRATE‐N (ver. 3.6.6, Beerli & Felsenstein, 1999, 2001). MIGRATE‐N enables the joint estimation of Θ and *M* in a matrix of populations from genetic data. Other software, such as BEAST (Drummond & Rambaut, 2007) ignores effects of migration which thus may be misinterpreted as changes in Θ (Heller et al., 2013). The most probable nucleotide mutation model and associated parameter values were selected using JMODELTEST (ver. 2, Darriba et al., 2012). The prior ranges of Θ and *M* were chosen from the results of preliminary MIGRATE‐N estimations at reduced sample sizes and Markov chain Monte Carlo (MCMC) lengths using the observed *F*_ST_ estimates, as starting values following the recommendations made by Beerli (2016). The specific analyses parameter values are tabulated in Table S1. The maximum sample size was set to 250 DNA sequences. For data sets with more than 250 DNA sequences. For larger data sets, 250 DNA sequences were selected at random (without replacement). Random sub‐sampling was also employed to reduce sample sizes to the smallest number in order to avoid biases due to uneven sample sizes (see the Supplementary Material “Notes on the effects of sample sizes” and Figure S1). Final estimates were obtained from three independent estimations, all initiated with different random seeds. Each estimation comprised 100 MCMC replicates, each, in turn, consisting of a single MCMC chain of 16 million steps, with the first eight million steps discarded as burn‐in and sampling at every 200th step. A static heating scheme was employed with four chains at temperatures of 1.0, 1.5, 3.0, and 1,000,000, respectively. Convergence was assessed with the R‐CRAN package *coda* (ver. 0.19‐1, Plummer et al., 2006). Consistency among the three separate MCMC estimates, along with smooth and unimodal distribution, within the prior range across all three estimations, was also viewed as indications of convergence. A minimum effective sample size of 10,000 was required for all parameter estimates. The final point estimates and the standard deviation of Θ and *M* were obtained by combining the three independent estimates (the three estimates are available in GitHub [see “Data Availability Statement”]). In order to account for differences in the number of MCMC data points employed to infer Θ and *M* in each time interval, weighted averages of the three medians and the standard deviations of Θ and *M* were estimated per time interval. The pooled medians of Θ and *M* were estimated as follows: ${\;m}_{p}=\sum_{i=1}^{k}n_{i}m_{i}/\sum_{i=1}^{k}n_{i}$, where, $n_{i}$ denotes the number of MCMC data points employed to estimate *i*. The term $m_{i}$ denotes the estimated median parameter value of estimate i, and *i* denotes the estimated parameter values of Θ or *M*. The term *k* denotes the total number of estimated parameter values (in this case, three independent MCMC estimates). The pooled standard deviation (${\mathrm{SD}}_{p}$) was estimated as ${\;\mathrm{SD}}_{p}=\sqrt{\sum_{i=1}^{k}\left(n_{i}‐1\right){{\mathrm{SD}}_{i}}^{2}/\left(\left(\sum_{i=1}^{k}n_{i}\right)‐k\right)}$, where ${\mathrm{SD}}_{i}$ denotes the estimated standard deviation of estimate i. The 95% confidence interval [CI] of the final estimates was approximated to ${m_{p}\pm 1.96\mathrm{SD}}_{p}/\sqrt{n_{i}}$(i.e., assuming a normal distribution of *m*).

Possible effects of intra‐oceanic, population genetic structure were assessed by comparing the outcome of estimates based on pooled and spatially distinct samples. Population samples were analyzed separately when there was a discernible effect of spatial population genetic structure on the final estimate (e.g., Northern krill). Following Sasaki et al. (2005), the two nominal right whale species were treated as different oceanic populations of a single species, due to their low degree of genetic divergence among oceanic populations of southern and North Atlantic right whales (Rosenbaum et al., 2000), which were similar to inter‐oceanic divergences estimated among con‐specific populations in other baleen whale species (e.g., Jackson et al., 2014).

Converting the time estimates obtained with MIGRATE‐N into calendar years necessitated estimates of generational mutation rates. A range of previously reported mutation rates (in the target or closely related species) was explored; the findings are described in the Supplementary Material “Notes on mutation rates,” Tables S2 and S3, and Figures S2 and S3. For the mitochondrial genome, the same generational mutation rate was assumed for all species. However, the final annual mutation rate differs among species because the generation times are species specific. In the case of the CR, a generational mutation rate at 1.125 × 10^−6^ per site was employed. This rate was within the range of previously reported estimates ranging from 2 × 10^−7^ to 2 × 10^−5^ per site per generation (Supplementary Material “Notes on mutation rates,” Figure S2). In the case of DNA sequences obtained from other mitochondrial genes (i.e., other than the CR), a generational mutation rate at 3.4 × 10^−7^ per site was employed. This value was within the range previously reported for the coding regions in the mitochondrial genome or the entire mitochondrial genome, 2 × 10^−8^–2 × 10^−4^ per site (Supplementary Material “Notes on mutation rates,” Figure S2).

The consistency among estimates obtained from DNA sequences collected from different regions of the mitochondrial genome was assessed by comparing the estimate of temporal changes in Θ inferred from different mitochondrial genes in the same species (Tables S1 and S4; Figure S3). Conspecific temporal changes in Θ, estimated from mtDNA sequences, were also compared to similar estimates obtained from genome‐wide, nuclear data produced by next generation sequencing in three selected baleen whale species (North Atlantic common minke whale, North Atlantic fin whale and southern right whale), as described below (see Section 2.4 below and Table S4; Figure S3).

### 2.4 Nuclear DNA data

#### 2.4.1 Laboratory methods

Genome‐wide single nucleotide polymorphisms (SNPs) were generated from sequencing double digested restriction‐associated (ddRAD, Peterson et al., 2012) and quadruple barcode ddRAD (quaddRAD) libraries (Franchini et al., 2017). Common minke whale and southern right whale libraries were generated from ddRAD libraries following Peterson et al. (2012). Fin whale libraries were generated from using the quaddRAD protocol as outlined by Franchini et al. (2017), which enables the removal of PCR clones. All libraries were prepared from genomic DNA digested with *Hind*III *and Msp*I and insert sizes between 300 and 400 bp. Libraries were sequenced on an Illumina HiSeq™ 2500 (ver. 4) as paired‐end sequencing at 100 (ddRAD) or 125 (quaddRAD) cycles, with 10% *Phi*X spike‐in.

#### 2.4.2 Data processing

In the case of the quaddRAD library, PCR clones were removed using the *clone_filter* script implemented in the software suite STACKS (ver. 1.47, Catchen et al., 2013). Low quality reads were removed from the raw FASTQ files with *process_radtags* (STACKS, ver. 1.47) using default settings. The output from process_radtags was concatenated in paired‐ and single‐end files for each species. The remaining reads were aligned to a reference genome with BOWTIE2 (ver. 2.2.8, Langmead & Salzberg, 2012) as an “end‐to‐end” alignment, employing the setting *very_sensitive* (i.e., *D* 20, *R* 3, *N* 0, *L* 20, and *i S*,1,0.50). The maximum fragment size for concordant paired‐end alignments was set at 600 and discordant alignments discarded. Reads from the common minke whale and fin whale were aligned against the common minke whale genome (Yim et al., 2014) and against the bowhead whale genome (Keane et al., 2015) in case of the southern right whale.

The folded site frequency spectrum (SFS, Fisher, 1930; Nielsen et al., 2012) was estimated using ANGSD (ver. 0.917, Korneliussen et al., 2014) from samples with a minimum of three million aligned reads. SNP genotype likelihoods from the mapped reads were estimated using the GATK model (McKenna et al., 2010) as implemented in ANGSD (ver. 0.917, Korneliussen et al., 2014). SNPs with a genotype quality below 20 and a mapping quality below 10 were discarded. Only SNPs genotyped in a minimum of 80% of the individuals were included in the estimation. For each species, two SFSs were estimated, using a minimum read coverage at 10 and 2, respectively. The two data sets differed in terms of the total number of SNPs (Table 1 and Supplementary Material “Notes on genome wide SNP genotype analyses”).

#### 2.4.3 Estimation of changes in genetic diversity from genome‐wide SNP genotypes

STAIRWAY PLOT (ver. 2.0 beta, Liu & Fu, 2015) was employed to infer temporal changes in Θ from the folded SFS estimated from the genome‐wide SNP data. An annual mutation rate at 1.07 × 10^−9^ per site (Yim et al., 2014) was employed to convert the estimates into years in all three baleen whale species. Generation times of 21.2 years for the common minke whale, 32.5 years for the fin whale, and 27.6 years for the southern right whale. These were the average of the estimates reported by Taylor et al. (2007) and Pacifici et al. (2013). Similar to the mitochondrial data, the temporal changes in genetic diversity (Θ) were employed as proxies in drawing inferences on changes in abundance.

The estimates of the demographic changes obtained from mitochondrial and nuclear data presented in this study were restricted to the period between 1 and 30 kya in order (i) to include the end of the LGM and the global warming during the Pleistocene–Holocene transition, and (ii) to exclude possible effects of more recent anthropogenic impacts, notably commercial whaling. The complete output of the demographic estimates is available on GitHub (see “Data Availability Statement”).

Due to the uncertainties surrounding determining mutation rates, we focused our interpretations on Θ rather than on the effective population size (*N*_e_). The latter is related to Θ and the mutation rate as follows: $N_{e}=\frac{\Theta }{4\mu }$, where μ denotes the generational mutation rate.

### 2.5 Temperature data

Surface air temperature (SAT) estimates for the Southern Hemisphere were inferred from deuterium measurements collected from the Antarctic EPICA Dome C Ice Core and obtained from Jouzel et al. (2007). For the Northern Hemisphere, continental atmospheric temperatures between 40 and 80°N (calibrated with oxygen isotope records from 57 sediment cores) were obtained from Bintanja et al. (2005).

### 2.6 Maps of ocean circulation, sea ice reconstructions and species ranges

Maps were generated with ARCGIS® (ver. 10.3; ESRI® Inc.). Ocean current data were obtained from the NOAA National Weather Service (2016). Contemporary and LGM permafrost and ground ice data were obtained from Brown et al. (2002) and Lindgren et al. (2016), respectively. Average sea ice extent during March for the Antarctic and September for the Arctic (in 2016) was obtained from the National Snow and Ice Data Center (Fetterer et al., 2017) and used as proxies for contemporary minimum annual sea ice extent in the south and north. Contemporary ice sheet and glacial projections were obtained from Natural Earth (2017). Antarctic summer sea ice coverage, ice sheet cover, and glacial extent during the LGM were obtained from Gersonde et al. (2005) and CLIMAP (1981). The data for the Arctic summer sea ice coverage, ice sheet, and glacial extent from the LGM were obtained from GLAMAP 2000 (Ehlers et al., 2011; Pflaumann et al., 2003).

The species ranges for baleen whales, Antarctic krill, and herring were obtained from the IUCN Red List (IUCN, 2012). Bowhead whale range was modified based on Baird and Bickham (2020). The species ranges for capelin and Northern krill were generated based on data from AQUAMAPS (Kaschner et al., 2016). Species ranges for copepods were generated based on Bonnet et al. (2005) and COPEPEDIA (ICES et al., 2017).

### 2.7 Correlations among baleen whales, prey, and climate

Pearson's correlation coefficients were estimated using R (ver. 3.2.5, R‐Development‐Core‐Team, 2016). Estimates of Θ and SAT were fitted to 1000‐year intervals by linear interpolation in R (ver. 3.2.5, R‐Development‐Core‐Team, 2016). Time intervals with missing data were excluded from the analyses. Consistency, and possible dependency, were evaluated by assessing correlations among different time interval ranges (i.e., at 1000, 2000, and 5000 years, respectively).

## 3 RESULTS

In total, 4761 mtDNA sequences from eight baleen whale species and 2271 mtDNA sequences from seven prey species (fish and invertebrates) were analyzed to infer temporal changes in Θ and M. In addition, between 14,304 and 62,579 genome‐wide SNPs were analyzed in a total of 100 individuals from three baleen whale species in order to assess if the temporal changes in Θ estimated from the mtDNA sequences likely reflected the genome‐wide genetic diversity (Table 1).

**FIGURE 3:**
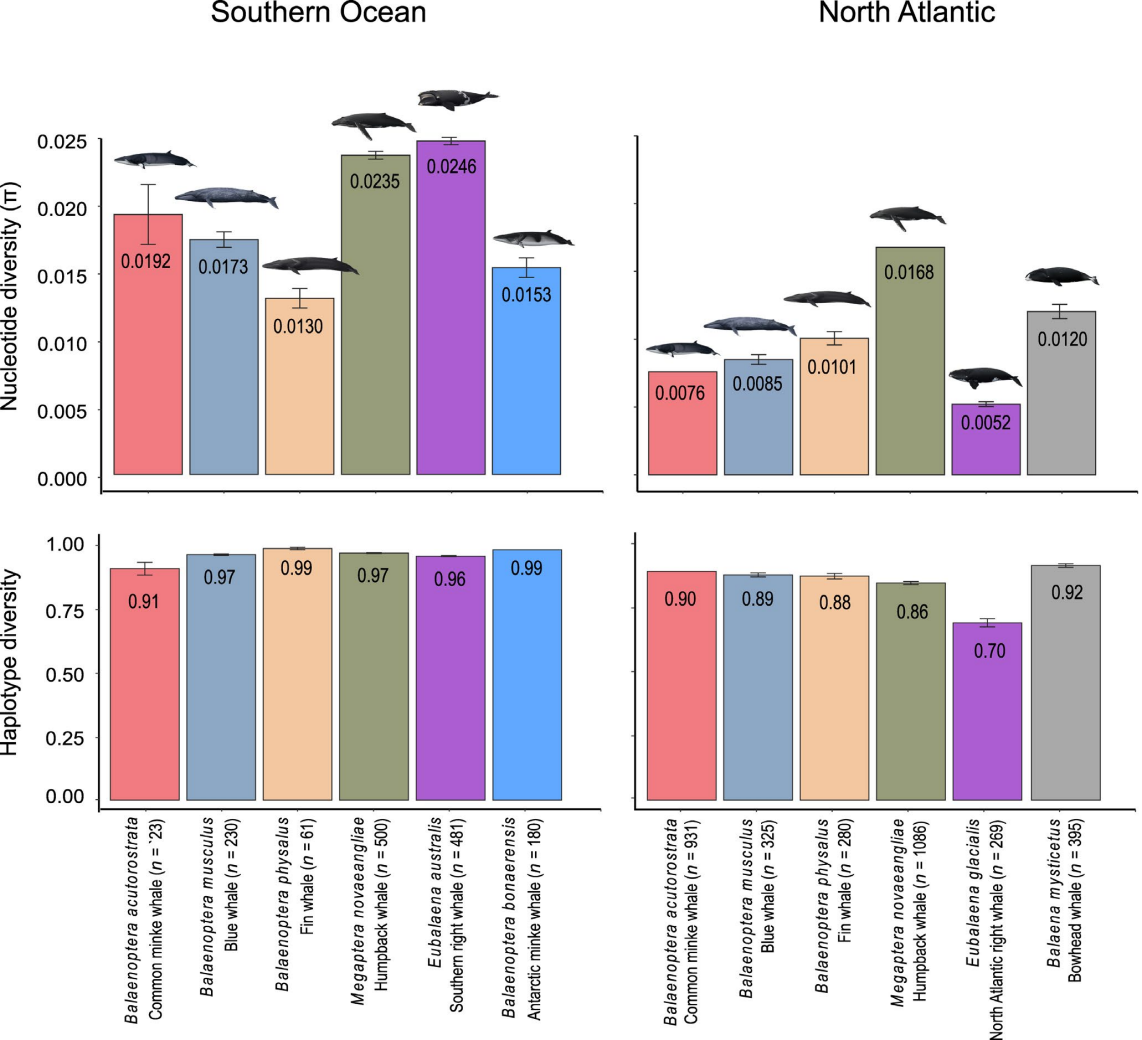
Haplotype and nucleotide diversity (*π*) from baleen whales of the Southern and North Atlantic oceans. Numbers represent mean estimates based upon mitochondrial control region DNA sequences. Sample sizes are listed in parentheses with the common species names

The mean genetic diversity was higher among the Southern Ocean baleen whales (*h*: .96, 95% CI: .91–.992; π: .0188, 95% CI: .0130–.0246, Figure 3) compared to baleen whales in the North Atlantic Ocean (*h*: .86, 95% CI: .70–.92; π: .0100, 95% CI: .0052–.0168, Figure 3). The estimates of common genetic diversity indices are tabulated in the Supplementary Material, Table S5.

**FIGURE 4:**
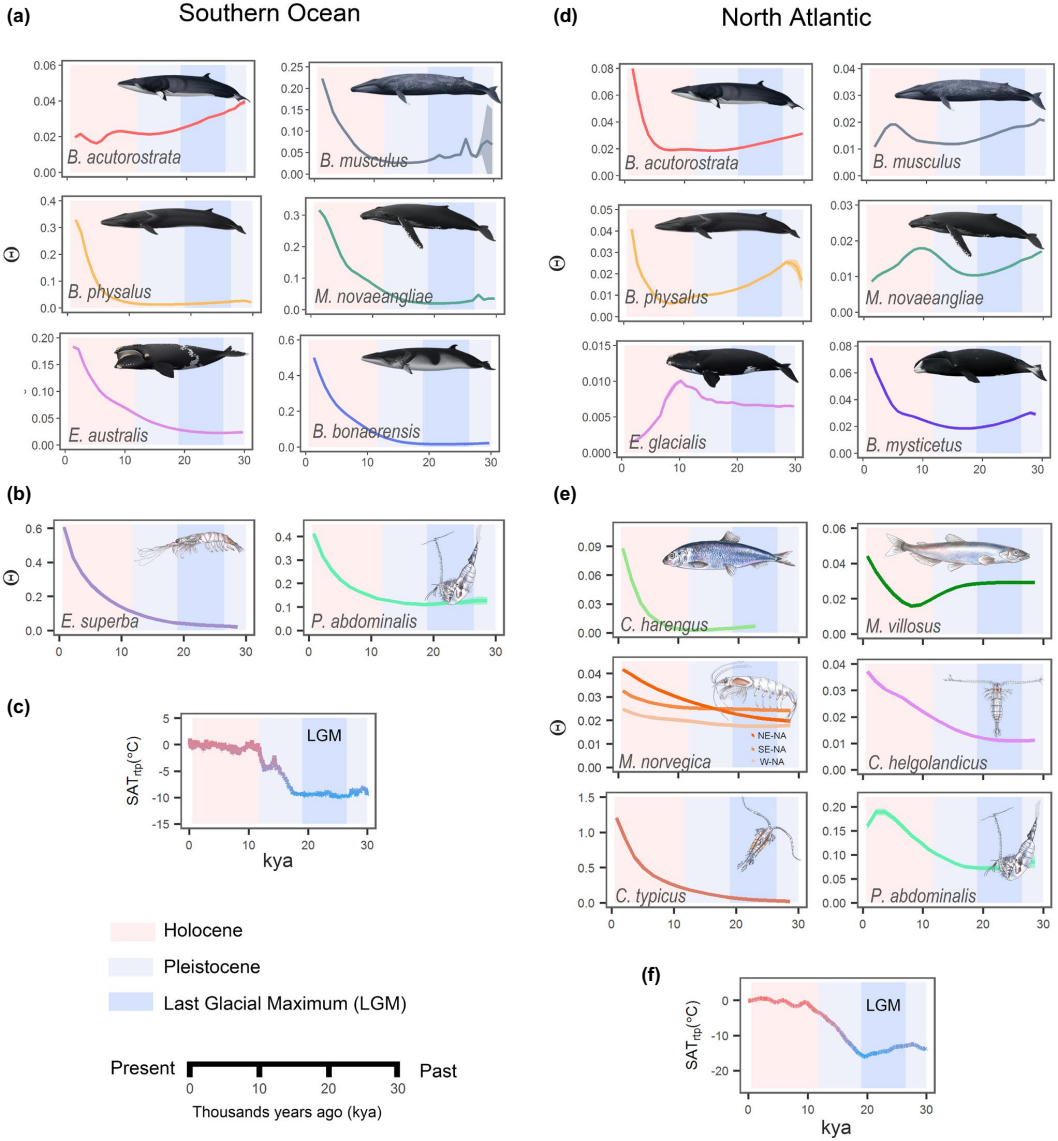
Temporal changes of Θ (a proxy for abundance inferred from the level of genetic diversity) during the Pleistocene and Holocene (1–30 kya). Temporal changes of in baleen whales and their prey in the Southern Ocean (a, b) and the North Atlantic Ocean (c, d). Note the different scales of the values on the vertical axis (Θ). Historical surface air temperature relative to present temperature (SAT_rtp_) in degrees Celsius (°C) in the Southern Ocean (c) and North Atlantic Ocean (f). Time estimates (in units of thousands of years ago, kya) along the horizontal axis. NE‐NA: northeastern North Atlantic (NA), SE‐NA: southeastern NA. W‐NA: western NA

**FIGURE 5:**
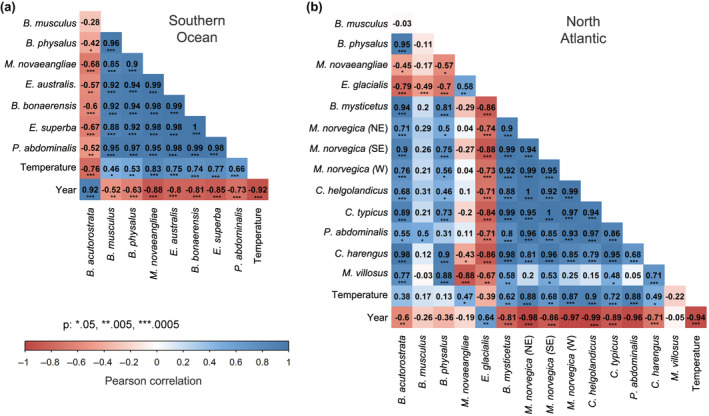
Pairwise Pearson’s correlations between Θ and temperature over time among baleen whales and their prey. (a) Southern Ocean and (b) North Atlantic Ocean. Blue: positive correlation, red: negative correlation. The interval between observations at 1000 years

The estimated temporal changes in Θ suggested that most baleen whale populations, irrespective of ocean basin, expanded when the global temperatures rose after the LGM, particularly during the Holocene (Figure 4). In the Southern Ocean, large, exponential, and synchronous increases were observed in Θ among all baleen whale species, except for the common minke whale (Figure 4a). Although the common minke whale underwent an initial decrease in Θ after the LGM, it subsequently underwent exponential increase beginning ~4 kya. The temporal trend of the increases in Θ was strongly and positively correlated among baleen whales (*r* = .84–.99, *p* < .0005, Figure 5a). Although similar, post‐LGM increases in Θ were also observed among the North Atlantic baleen whale species (Figure 4d), the specific timing and duration of the increase varied considerably. The blue, humpback, and the North Atlantic right whale all underwent an initial post‐LGM increase followed by a subsequent decline 6–10 kya. In contrast, the exponential increases in Θ observed in the fin, common minke, and bowhead whale were delayed until 6–8 kya (Figure 4d).

Temporal changes in Θ in key prey species, such as krill, copepods, capelin, and herring were estimated as well in order to assess if the post‐LGM changes in Θ observed among the baleen whales were correlated with prey availability (Figure 2g–l, Table 1). In the Southern Ocean, large exponential and synchronous increases in Θ were estimated in Antarctic krill and *P*. *abdominalis*, starting around 15–23 kya (Figure 4b). These increases were congruent with the increases observed among the baleen whales in the Southern Ocean. As was the case for the baleen whales, the temporal changes in Θ varied considerably among the prey species in the North Atlantic Ocean. An increase in Θ immediately after the LGM was observed in all invertebrates (i.e., Northern krill, and three copepods, *Ca*. *helgolandicus*, *Ce*. *typicus*, and *P*. *abdominalis*), whereas the estimated increases in Θ in fishes (capelin and herring) were observed 6–8 kya (Figure 4e). In the Southern Ocean, significant and strong correlations (*r* = .88–1.00, *p* < .0005) were observed between the krill foraging baleen whales (i.e., the Antarctic minke, southern right, humpback, blue and fin whale) and the two prey species (i.e., Antarctic krill and *P*. *abdominalis*, Figure 5a). The common minke whale was the only species in the Southern Ocean (*r* = −.33–.30, Figure 5a) in which no statistically significant correlation was observed with Θ in other baleen whale or prey species. Among the North Atlantic baleen whales, strong, positive correlations in the temporal changes of Θ were observed among the fin, the common minke, and the bowhead whale (*r* = .69–.92, *p* < .0005, Figure 5b), whereas the correlation was significant, but slightly weaker, between the humpback and right whale (*r* = .64, *p* < .005). Among the North Atlantic prey species, krill and copepod species showed the strongest positive correlations (*r* = .85–1.00, *p* < .005). The strongest positive correlations between North Atlantic baleen whale and prey species (*r* = .46–.99, *p* < .05) were observed for the common minke, fin, and bowhead whale. Varying the time intervals between observations from every 1000 years to every 2000 and 5000 years had little or no effect on the estimated correlations (Figure S5).

**FIGURE 6:**
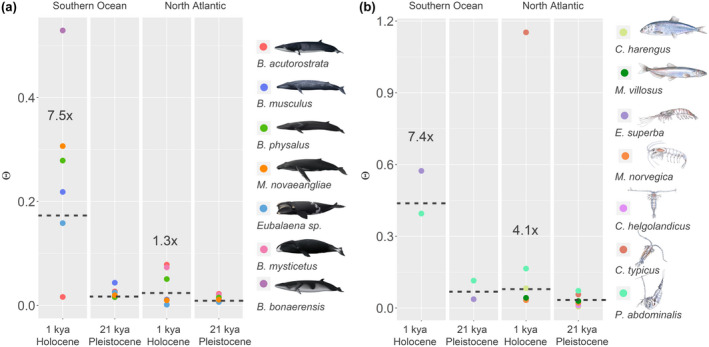
The relative change in abundance (ΔΘ) in baleen whales and their prey during the Pleistocene and Holocene. Circles represent the median point estimates of Θ for each species of baleen whales (a) and prey (b). Dotted lines denote the geometric mean of Θ (estimated from all point estimates). A “×” (e.g., 7.5×) denotes the relative change in Θ (ΔΘ) at one thousand years ago (kya) relative to 21 kya

The estimated average net changes in Θ (denoted ΔΘ) after the LGM (~21 kya) and late Holocene (~1 kya) were employed as a means to assess the intensity of the global warming on the abundance of baleen whale and prey species in the Southern and North Atlantic Ocean. Estimates of ΔΘ were higher for baleen whales in the Southern Ocean relative to the North Atlantic Ocean (Mann‐Whitney test, *U* = 3, *n*_1_ = 5, *n*_2_ = 5, *p* < .05) with averages at 9.0 (range: 1.3–34) and 1.2 (range: 0.3–3.6), respectively (Figure 6a). A similar, albeit less pronounced, difference in average ΔΘ was observed among the prey species between the two oceans, which was estimated at 7.4 (range: 3.5–15.7) in the Southern Ocean and 4.1 (range: 1.5–20.4) in the North Atlantic Ocean (Figure 6b).

**FIGURE 7:**
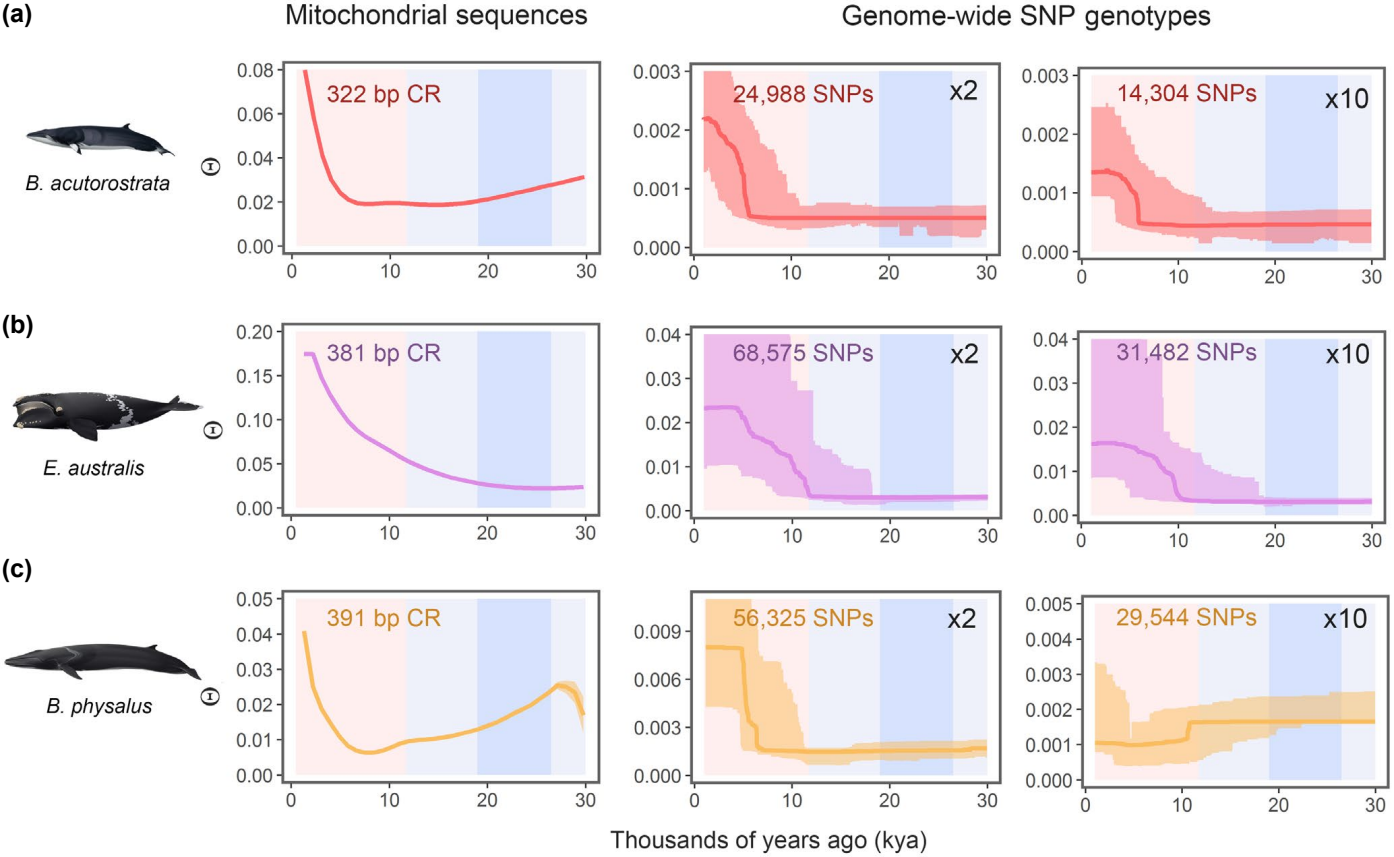
Temporal changes in Θ estimated from mtDNA control region sequences and genome‐wide SNP genotypes of two levels of minimum read depth. Estimated demographic history employing mtDNA control region and genome‐wide SNP genotypes (at a minimum coverage at ×2 and ×10, respectively) in North Atlantic common minke whale (a), North Atlantic fin whale (b) and southern right whale (c). Time in thousands of years ago (kya) is along the horizontal axis, the estimates of Θ are along the vertical axis. Red and blue shading denotes the Holocene and Pleistocene, respectively. The darkest blue indicates the LGM

The temporal changes in Θ inferred from the genome‐wide SNP data in three selected baleen whale species were consistent with and gave support to those inferred from the mtDNA sequence variation (Figure 7), that is, baleen whale population expansions during the Pleistocene–Holocene transition (see Supplementary Material “Notes on genome‐wide SNP genotype analyses”). Increasing the read depth of the genome from ×2 to ×10 reduced the number of SNPs by ~50% (Table 1), which in turn muted the degree of the estimated expansions, likely an effect of removing rare variants. The estimated temporal changes in Θ were consistent with the changes estimated from mtDNA sequences in the common minke and the southern right whale at both read depths. While in the fin whale, the median estimate of the temporal changes in Θ did not indicate an exponential expansion at a read depth at ×10, the 95% confidence bands at both read depths were nearly identical (Figure 7; Figure S4).

The estimates of M (i.e., inter‐ocean basin connectivity) were subject to substantial uncertainty (Figure S6). Overall, the inter‐oceanic intra‐specific connectivity in baleen whales increased during two periods: (i) after the population expansions observed in the Southern Hemisphere during the Pleistocene–Holocene (Figure 4) and (ii) during the LGM, when the range for most baleen whales was contracted toward the Equator due to an increase in polar sea ice extent in both hemispheres (Figure 1c–f), thus placing conspecific oceanic populations in closer proximity.

## 4 DISCUSSION

### 4.1 Impacts of past global warming

The Pleistocene–Holocene transition after the LGM represented a period when global temperatures rose rapidly, which, in turn, led to drastic reductions in polar sea ice extent and substantial sea level rises (Abrantes, 2000; Clark et al., 2009). The net result of these oceanographic changes was an overall expansion of suitable marine habitat and concurrent, large‐scale changes in ocean circulation that resulted in increased primary productivity (Tsandev et al., 2008). These changes were not instantaneous but rather they were set in motion, creating a long‐term re‐configuration of the marine environment that affected marine and terrestrial flora and fauna throughout the Holocene. The present study revealed ocean‐wide increases in baleen whale abundance (here represented by Θ) in both the Southern Ocean and North Atlantic during this period of global warming. The expansions in baleen whale abundances were correlated with temperature changes and concomitant increases in the abundances of their prey. The rate of increase in abundance peaked during the early Holocene and appears to be a general, and previously unrecognized, feature of both ocean basins.

Subsequent changes in the rate of increase or decrease of baleen whale abundance were observed mostly in the North Atlantic Ocean. These differences were consistent with the large‐scale dissimilarities in oceanographic features between the North Atlantic Ocean and the Southern Ocean (Moline et al., 2008). The Southern Ocean is dominated by a single, major and persistent current; the Antarctic Circumpolar Current (Tynan, 1998). In contrast, the North Atlantic Ocean is influenced by several smaller, less stable, interacting cold and warm water currents as well as periodic climate oscillations (O'Hare et al., 2005; Rossby, 1996). The Southern Ocean pelagic food web is also based mostly on Antarctic krill (Hopkins, 1985), whereas the North Atlantic Ocean pelagic food web basis comprises a diverse zooplankton community subject to fluctuations in abundances, in part due to the abovementioned periodic climate oscillations (Pershing et al., 2005). The large and exponential increase in Antarctic krill, the prey base for baleen whales in the Southern Ocean, would explain the larger baleen whale populations in the Southern Ocean and the largely identical and synchronous increase in abundance among baleen whales during the post‐LGM global warming. In contrast, the prey base and environmental conditions in the North Atlantic Ocean varied considerably over time and space (see below), which is reflected in the absence of a similar synchronous change in baleen whale abundance in this ocean basin.

The temporal trend in the rate of increase or decrease observed among North Atlantic baleen whales and their prey species appeared to change substantially 6–10 kya; either transitioning from an increase to a decrease in abundance or from a stable to an exponentially increasing abundance. The so‐called “8.2 kya event” (Alley et al., 1997) took place during this epoch. The 8.2 kya event was caused by a massive discharge of glacial melt water that was released into the western North Atlantic Ocean from proglacial lakes, which led to a precipitous drop in global ocean temperatures (Alley et al., 1997; Barber et al., 1999). Sediment core diatom records suggest that the 8.2 kya event resulted in a major shift in phytoplankton composition consistent with a reduction in primary productivity, in particular in the western North Atlantic Ocean (Caissie et al., 2010; Harland et al., 2016). The decline in primary productivity could have led to a reduction of prey and deteriorating environmental conditions, possibly leading to declines in some baleen whale populations. However, apart from the copepod *P*. *abdominalis*, the few North Atlantic prey species included in this study did not appear to have declined in abundance after the 8.2 kya event.

Samples from the herring originated mostly from the Baltic Sea. Due to potential founder effects, the demographic history of Baltic Sea populations might differ from those of the North Atlantic. Additional sampling across space and additional species at lower trophic levels may yield further insights. The level of inter‐specific competition among the baleen whales or competition with other marine mammals may also have been affected by the concomitant environmental changes due to the 8.2 kya event in turn leading to changes in abundance.

The temporal differences in the rate of change in abundance observed between most Southern Ocean baleen whale and the common minke whale most likely stem from ecological differences. Common minke whales are distributed at comparatively lower latitudes and feed on myctophid fishes (Kato & Fujise, 2000). In contrast, the other Southern Ocean baleen whale species in this study, occupy higher latitudes, closer to the sea ice where they feed primarily on krill (Kawamura, 1980).

The strong correlation observed in the changes in abundance between baleen whale species and their prey, for example, between the krill‐eating baleen whales and Antarctic krill, suggest that baleen whales responded in synchrony to ocean‐wide ecological changes, such as prey availability (Ims & Andreassen, 2000; Korpimӓki et al., 2005; Seyboth et al., 2016), geological conditions (Hansen et al., 2013), or a combination of the two. Similar post‐LGM increases have been observed in krill‐eating Antarctic penguin species (Frugone et al., 2018; Trucchi et al., 2014; Younger, Clucas, 2015; Younger, Emmerson, 2015), suggesting that the apparent, large increase in primary productivity was sufficient to drive expansions in abundances across all krill predators.

Numerous studies, either at local or regional scales, have proposed that retreating polar sea ice extent and upwelling led to an increase in the abundance of key zooplankton species, such as krill, copepods, and other meso‐zooplankton species (Loeb et al., 1997; Moore, 2016; Stenseth et al., 2002). However, to the best of our knowledge, this study is the first to conduct a global assessment at such an extensive temporal scale. The results documented a global‐scale expansion in baleen whale prey availability, which began at the end of the Pleistocene and continued throughout the Holocene.

**FIGURE 8:**
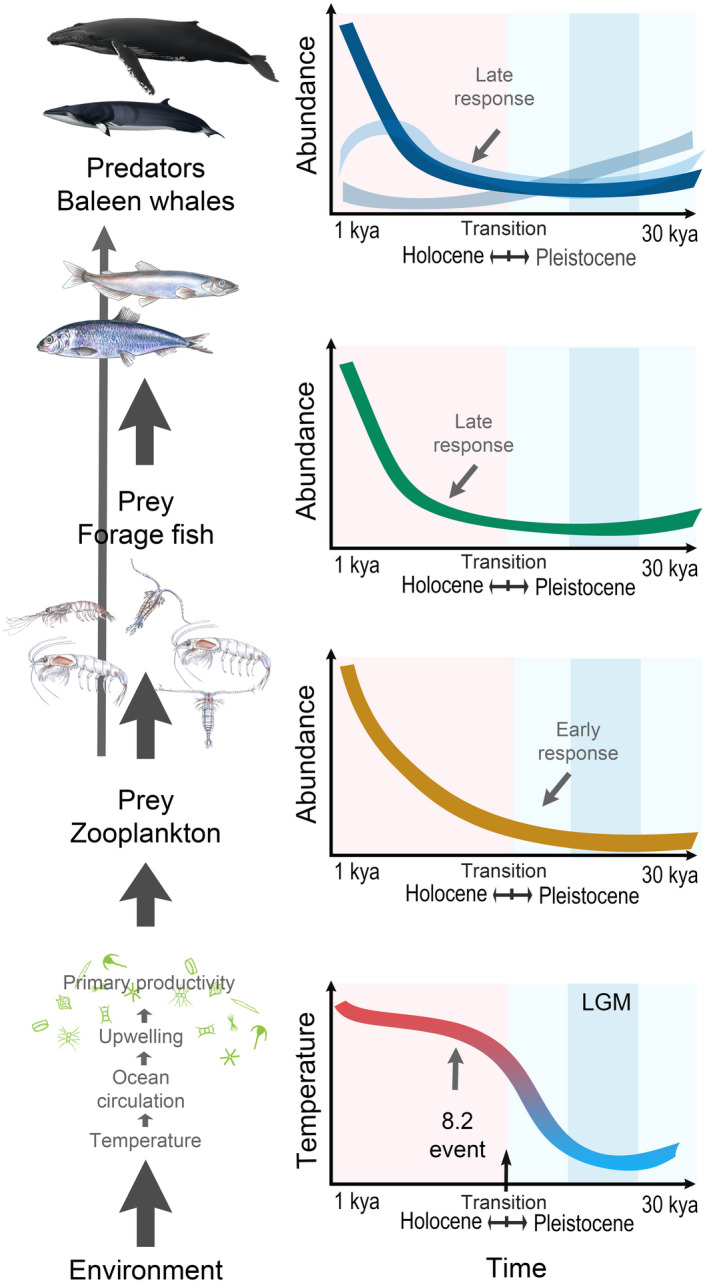
Bottom‐up control model of the demographic responses of baleen whales during the Pleistocene‐Holocene transition. Red and blue shading denotes the Holocene and Pleistocene, respectively. The darkest blue shading defines the LGM

The question whether ecosystems are primarily bottom‐up or top‐down controlled is a fundamental, debated topic in ecology, particularly in marine ecosystems. Our results supported bottom‐up control of the pelagic ecosystems during the last 30 ky. The post‐LGM increases in abundance at the lower trophic levels (e.g., krill and copepods) appeared to precede the increases in abundance at the higher trophic levels (e.g., fishes and baleen whales), although the precision of the exact temporal placement within and among species makes it difficult to draw affirmative conclusions. This initial increase in prey abundance was observed in both ocean basins, suggesting a bottom‐up (White, 1978) enrichment of the oceans during the initial warming phase during the Pleistocene–Holocene transition (Figure 8). This is consistent with previous paleo‐oceanographic modeling (Radi & de Vernal, 2008; Tsandev et al., 2008) and a shift in phytoplankton composition from perennial pelagic to seasonal sea‐ice‐associated species during the Pleistocene–Holocene transition. The latter species are indicative of high levels of primary productivity (Caissie et al., 2010; Harland et al., 2016).

### 4.2 Critical evaluation of methods

We assessed the impact of employing different mitochondrial genes and compared the results from mitochondrial genes with genome‐wide SNPs in a subset of species. The results from the different genes (Figure S3), or between the nuclear and mitochondrial genome (Figure 7), were congruent in terms of the long‐term demographic trends. This outcome suggests that the inferences drawn from single‐locus mitochondrial genes were robust, which is unsurprising given the long time and large spatial scale of the estimations.

A relevant, but unavoidable caveat to this study, as well as similar assessments based on genetic inference methods, is the inherent uncertainty of the applied mutation rates, which is consequential in terms of inferring the timing of changes in Θ and *M*. Although the choice of mutation rates has implications for placing the detected changes at a specific annual time, the demographic trends are unaffected to the choice of mutation rate. In this study, the chosen generational mutation rates were within the range of published generational mutation rate estimates in vertebrate and invertebrate species, such as baleen whales, fish, and crustaceans (Supplementary Material “Notes on mutation rates,” Tables S2 and S3). However, the possibility that the annual estimates are biased cannot be excluded in this or any similar genetic assessments. Nevertheless, the overall consistency observed in this study among the annual estimates based upon different mitochondrial regions with those estimates obtained from genome‐wide SNPs (employing different mutation rates) was reassuring.

Many and different processes affect demographic change, such as fluctuations in habitat and resource availability (e.g., Foote et al., 2013), prey preference (e.g., Fleming et al., 2016; Víkingsson et al., 2014), as well as inter‐ and intra‐specific competition (e.g., Moore, 2016; Moore et al., 2019). Disentangling the relative contributions of all the processes that may contribute to the observed temporal and spatial trends in demographic changes is non‐trivial, and will require substantial efforts. The strength of this study lies with the overall sampling design, which includes extensive horizontal (i.e., same trophic level) and vertical (i.e., prey and predators) ecological sampling. The inclusion of multiple species can be viewed as "pseudo‐replication": in effect each species, at a given trophic level, represents a single response to the underlying, global processes, such as global warming. The overall consistency in the observed demographic trends across multiple taxa and trophic levels suggests that our analysis captured fundamental drivers of change.

### 4.3 Implication for future global warming

The rapid rise in global temperatures during the Pleistocene–Holocene transition plateaued ~10 kya, yet most vertebrate and invertebrate taxa included in this study continued to increase in abundance in both ocean basins (until ~1 kya, the most recent time point included in this analysis). In other words, the Pleistocene–Holocene transition appeared set into motion long‐term oceanographic and ecological transitions that continued to change abundance and connectivity among baleen whales and their prey during an additional ~10 ky. This observation raises the possibility that the current global warming has already set processes in motion that will result in long‐term and wide‐ranging rearrangements in marine ecosystems that will continue for millennia after temperatures stabilize. The stabilization of global temperatures during the Holocene may also be a subsequent contributing factor, although exactly how constancy per se would facilitate change (here in abundance and migration rates) is not readily evident. Global warming impacts a wide variety of known and unknown processes; abiotic (e.g., the 8.2 kya event) and biotic, which in turn possibly interact as well. The analysis presented here provides a broad picture, but is insufficient to discern among the many more detailed plausible interactions and processes.

A number of recent studies have attempted to predict the effects of current global warming on marine mammals from contemporary, short‐term field observations, and known aspects of each species' ecology (Laidre et al., 2008; Moore & Huntington, 2008; Tulloch et al., 2019). Some baleen whale populations, such as humpback, fin, and blue whales, appear to arrive earlier on the summer foraging grounds (Ramp et al., 2015) and at increasingly higher latitudes, potentially increasing competition with obligate polar species, such as the bowhead whale (Moore, 2016; Moore et al., 2019). Similarly, changes in the distribution and migratory routes linked to sea ice concentrations (Druckenmiller et al., 2018), plasticity in diet preferences associated with changes at lower tropic levels (Fortune et al., 2020), and behavioral adaptations due to increasing killer whale predation (Matthews et al., 2020) have been observed in bowhead whales. Although the findings reported here suggested that most baleen whale species may benefit from the global warming, the results also suggest that the effects of increasing temperatures on baleen whale abundance and migration are complex, with potential wide ranging and long‐lasting impacts. In addition, the rate of current on‐going global warming is higher than the post‐LGM warming and will likely reaching higher temperatures (Bova et al., 2021; Foster et al., 2017). Regional oceanographic conditions, including temperature, annual sea ice dynamics, and prey base, appear to have been the main driver of the long‐term responses seen in baleen whales during past epochs characterized by global warming. However, the rapid and extreme sea ice loss, predicted in the coming decades due to current global warming, including unprecedented ice‐free summers (which did not apply to the Pleistocene–Holocene transition) leaves considerable uncertainty as to the future of all whales.

## CONFLICT OF INTEREST

The authors declare no competing interests.

## AUTHOR CONTRIBUTIONS

Per J. Palsbøll and Andrea A. Cabrera conceived and designed the study with input from Elena Schall. Pia Anderwald, Lutz Bachmann, Simon Berrow, Peter B. Best, Phillip J. Clapham, Haydée A. Cunha, Luciano Dalla Rosa, Kenneth P. Findlay, Tore Haug, Mads Peter Heide‐Jørgensen, A. Rus Hoelzel, Kit M. Kovacs, Scott Landry, Finn Larsen, Christian Lydersen, David K. Mattila, Richard M. Pace III, Chiara Papetti, Luis A. Pastene, Christian Ramp, Jooke Robbins, Richard Sears, Eduardo R. Secchi, Mónica A. Silva, Rui Prieto, Gísli Víkingsson, Øystein Wiig, and Nils Øien provided data or materials. Martine Bérubé, Tom Oosting, and Carolina Dias conducted laboratory analyses. Andrea A. Cabrera, Elena Schall, and Xênia Moreira Lopes conducted data analyses supervised by Per J. Palsbøll and with input from Angeliki Paspati. Andrea A. Cabrera designed the figures. Andrea A. Cabrera and Per J. Palsbøll drafted the manuscript with contributions from Martine Bérubé, Elena Schall, Jooke Robbins, Christian Ramp, Phillip J. Clapham, Xênia Moreira Lopes, and Kit M. Kovacs. All authors read, edited, commented on, and approved the final manuscript;

## Supporting information

Supplementary Material

## Data Availability

Mitochondrial and nuclear data information, analysis scripts, input files, analysis outputs, and other relevant data have been deposited at GitHub (https://doi.org/10.5281/zenodo.5838316). Mitochondrial sequence data can be found in the input files for Migrate analyses. Raw FASTQ files from the nuclear data have been deposited in NCBI's Sequence Read Archive (Bioproject ID: PRJNA791939; Sample ID: SAMN24369310‐SAMN24369409; http://www.ncbi.nlm.nih.gov/bioproject/791939).
