# Supplementary material for: Strong and lasting impacts of past global warming on baleen whales and their prey
Source: Glob Chang Biol. 2022 Feb 2;28(8):2657–77. doi: 10.1111/gcb.16085 (PMC9305191; doi:10.1111/gcb.16085)
Supplement: Supplementary file 1 — Supplementary Material [file GCB-28-2657-s001.docx]

Supplementary Materials for

**Strong and lasting impacts of past global warming on baleen whales and their prey**

Andrea A. Cabrera, Elena Schall, Martine Bérubé, Pia Anderwald, Lutz Bachmann, Simon Berrow, Peter B. Best, Phillip J. Clapham, Haydée A. Cunha, Luciano Dalla Rosa, Carolina Dias, Kenneth P. Findlay, Tore Haug, Mads Peter Heide-Jørgensen, A. Rus Hoelzel, Kit M. Kovacs, Scott Landry, Finn Larsen, Xênia Moreira Lopes, Christian Lydersen, David K. Mattila, Tom Oosting, Richard M. Pace III, Chiara Papetti, Angeliki Paspati, Luis A. Pastene, Rui Prieto, Christian Ramp, Jooke Robbins, Richard Sears, Eduardo R. Secchi, Mónica A. Silva, Malene Simon, Gísli Víkingsson, Øystein Wiig, Nils Øien and Per J. Palsbøll

Correspondence to: Andrea A. Cabrera (andrea_ca_gt@yahoo.com) and Per J. Palsbøll (palsboll@gmail.com).

**Supplementary material includes:**

Notes on the effects of sample sizes

Notes on mutation rates

Notes on genome-wide SNP genotype analyses

Figures S1 to S6

Tables S1 to S5

Notes on the effects of sample sizes

The results obtained from the mitochondrial DNA (mtDNA) sequences in six baleen whales were compared between two different sample sizes per sample partition (i.e., 250 and 23, Figure S1). In the first case, random sub-sampling was set at 250 mitochondrial DNA sequences per sample partition which resulted in unequal sample sizes among sample partitions in some species (Figure S1A). In the three species with unequal sample sizes, two species contained sample partitions that differed by more than 100% (common minke whale, *Balaenoptera acutorostrata*, and fin whale, *Balaenoptera physalus*). In the second case, a second random sub-sampling was undertaken to compare the results obtained at equal sample sizes (i.e., the size of the smallest sample partition, in this case n = 23) with the larger, but unequal sample sizes.

In general, the changes in $\Theta$ based on a random sub-sampling at a sample size of 250 mtDNA sequences per sample, were similar to those estimated with random sub-sampling at 23 mitochondrial DNA sequences per sample (Figure S1). The agreement in parameter estimates between large and small sample sizes in coalescent-based approaches has been noted previously by Plutzhnikov and Donnelly (1996) and Felsenstein (2006) who concluded that even very low sample sizes (n = 8) were sufficient to accurately infer $\Theta$ in a single isolated population with constant population size. However, larger sample sizes are likely required in the presence of migration or population size changes (Pluzhnikov & Donnelly, 1996).

Low levels of variation were observed in the common minke whale. The median estimate of the changes in $\Theta$ in the presence of unequal sample sizes indicated a smaller change in $\Theta$ towards the present (Figure S1A) compared to the estimate obtained with lower but equal sample sizes (Figure S1B). Coalescent-based approaches rely on sampling across the entire parameter space during the MCMC estimation. Unequal sample sizes may result in under-sampling from partitions with low sample sizes when the number of MCMC steps is insufficient (Beerli, 2009). The gain from large sample sizes appeared to be offset by poorly evaluated parameter estimates, due to unequal sample sizes, and hence favored a sampling strategy with lower, but equal, sample sizes.

Notes on mutation rates

*Choice of mutation rate.* The estimates of time (𝜏) obtained from the coalescent-based approach employed in this study are scaled by the generational mutation rate per nucleotide site ($\mu$). Consequently, a conversion of 𝜏 into years ($t$) was necessary in order to relate the estimated changes in $\Theta$ to environmental change, such as increasing global temperatures. Such conversion required an estimate of the generation time or an annual mutation rate per site for the DNA sequences from which 𝜏 was estimated. The estimates of changes in abundance, or more precisely the effective population size (*N*_e_), were inferred from$\Theta(=4N_{e}\mu)$ as the focus was on relative (as opposed to absolute) change in *N*_e_. Relying upon $\Theta$ assumes that $\mu$ is constant, a common assumption, which some authors have argued may not necessarily hold true (Ho & Larson, 2006; Ho et al., 2005).

Mutation rates vary considerably among different DNA sequences across a genome (Hodgkinson & Eyre-Walker, 2011), as well as among genomes and species (Nabholz et al., 2009). Additionally, reported mutation rates also differ among estimation methods by as much as an order of magnitude (e.g., Ho et al., 2007; Howell et al., 2003; Rooney et al., 2001). Examples of published mutation rate estimates based upon coding and non-coding mtDNA sequences from species targeted in this study (or from the same taxonomic group when the targeted species were not available) are listed in Table S2 and Table S3.

In this study, two different generational mutation rates were applied, one for coding and one for non-coding DNA sequences (Aquadro & Greenberg, 1983). These two generational mutation rates were applied across all species. In terms of the annual mutation rate, the consequence of this choice implied that species with short and long generation times have high and low annual mutation rates, respectively. The consistency of the choice of the generational mutation rates for non-coding and coding DNA sequences was corroborated by comparing estimates of the change in $\theta$ based on from different regions in the same species (Figure S2C), although such an assessment does not negate the possibility of a systematic bias.

*Choices of mtDNA mutation rates.* The estimates in this study were based upon sequence variation in non-coding and coding mtDNA sequences. The specific choice of DNA sequences was determined by data availability (published or new data). All mtDNA data from the baleen whales were from the non-coding control region. The mtDNA sequence data from fish and invertebrate species were from published sources and all from coding mtDNA sequences, such as cytochrome *c* oxidase subunit I (COI), NADH dehydrogenase subunit 1 (ND1), cytochrome *b* (CYTB) or 16S ribosomal DNA (16S rDNA).

As mentioned above, different generational mutation rates were applied for coding and non-coding mtDNA (Aquadro & Greenberg, 1983). The specific mutation rates were chosen from species with well-characterized generation times and available estimates of annual mutation rates or from species from which estimates of the generational mutation rate were available. Among marine fishes and invertebrates, it has been commonplace to employ annual mutation rates that use the rise of the Central American Isthmus as the temporal calibration point (Lessios, 2008). However, recent findings have suggested that the previously recognized timing of the closure of the Central American Isthmus may be incorrect (Bacon et al., 2015; Montes et al., 2015), and hence mtDNA mutation rate estimates based solely upon this single calibration point were excluded. In the case of the coding mtDNA sequences, the applied mutation rate was selected from those species where an average mutation rate for multiple coding mtDNA sequences was available, i.e., excluding estimates based upon a single coding mtDNA sequence or the entire mitochondrial genome (i.e., including the faster evolving non-coding DNA sequences).

*Specific mutation rates.* Following the rationale outlined in the above sections, specific mutation rates were chosen as described below. One generational mutation rate of 1.12x10^-6^ per base pair (bp) was applied to all baleen whale estimations based upon mtDNA control region sequences. This value was obtained from the annual mutation rate estimated from minke whale mtDNA control region sequences, i.e., 5.3 x 10^-8^ per bp (Alter & Palumbi, 2009) and a generation time of 21.2 years (Pacifici et al., 2013; Taylor et al., 2007). This rate was within the range of previously applied values (from 2 x 10^-7^ to 2 x 10^-5^ per bp, median value: 1.24 x 10^-6^ per bp, Table S2, Figure S2A).

Similarly, one generational mutation rate (3.4 x 10^-7^ per bp) for coding mtDNA sequences was applied to all invertebrate and vertebrate prey species. The specific rate applied was based upon an estimated annual mutation rate at 1.7 x 10^-8^ for the coding mtDNA sequences in the human mitochondrial genome reported by Ingman *et al*. (2000) and a human generation time of 20 years (Nachman & Crowell, 2000). This rate was within previously reported values from vertebrate and invertebrate species (from 2.5 x 10^-8^ to 1.2 x 10^-6^ (with one exception at 2.0 x 10^-4^ per bp), median value: 1.03 x 10^-7^, Table S3, Figure S2B).

The consistency of the estimated changes and time points of change in $\Theta$ between non-coding and coding mtDNA sequences was assessed by comparing estimates from non-coding and coding mtDNA in North Atlantic herring (*Clupea harengus*) for which such data were available (Table S1 and Table S4, Figure S3). As expected, the estimates were consistent among non-coding and coding mtDNA using the applied annual mutation rates (Figure S3).

Notes on genome-wide SNP genotype analyses

The results obtained from the mtDNA sequences in three baleen whales were corroborated with similar estimates of the changes in $\Theta$ based upon genome-wide SNP genotypes in a total of 100 baleen whale specimens across three species (Table 1). The changes in $\theta$ based on the genome-wide SNP genotypes were consistent with those estimated from mtDNA sequence variation (Figure S3 and S4). The changes of $\Theta$ estimated from mtDNA sequences and the x2 coverage genome-wide SNP genotypes all suggested population expansions during the Pleistocene-Holocene transition in the three baleen whale species (i.e., the North Atlantic common minke whale, the fin whale, and the southern right whale, *Eubalaena australis*).

Increasing the read depth from x2 to x10 but reducing the number of SNPs, resulted in a less dramatic expansion (Figure S4). However, the changes of$\Theta$remained the same for the common minke whale and the southern right whale at both levels of coverage. In the case of the fin whale, the median estimates of the changes in $\Theta$ at a coverage of x10 did not indicate a population expansion (Figure S4). However, although the median estimate did not indicate a population expansion, the confidence band was very similar to the confidence band inferred at a coverage at x2. Detection of recent expansions relies upon the presence of rare alleles, which in turn requires a large number of SNP genotypes and large sample sizes. A recent simulation-based evaluation demonstrated an inability of “stairway plots” to detect recent population expansions (i.e., <10 kya) when sample sizes were small, i.e., ~ 30 samples (Liu & Fu, 2015). The sample sizes employed in this study were 27, 45 and 28 samples for the common minke whale, southern right whale and fin whale, respectively. This suggests that a recent expansion cannot be excluded, the expansion could have taken place but was undetected.


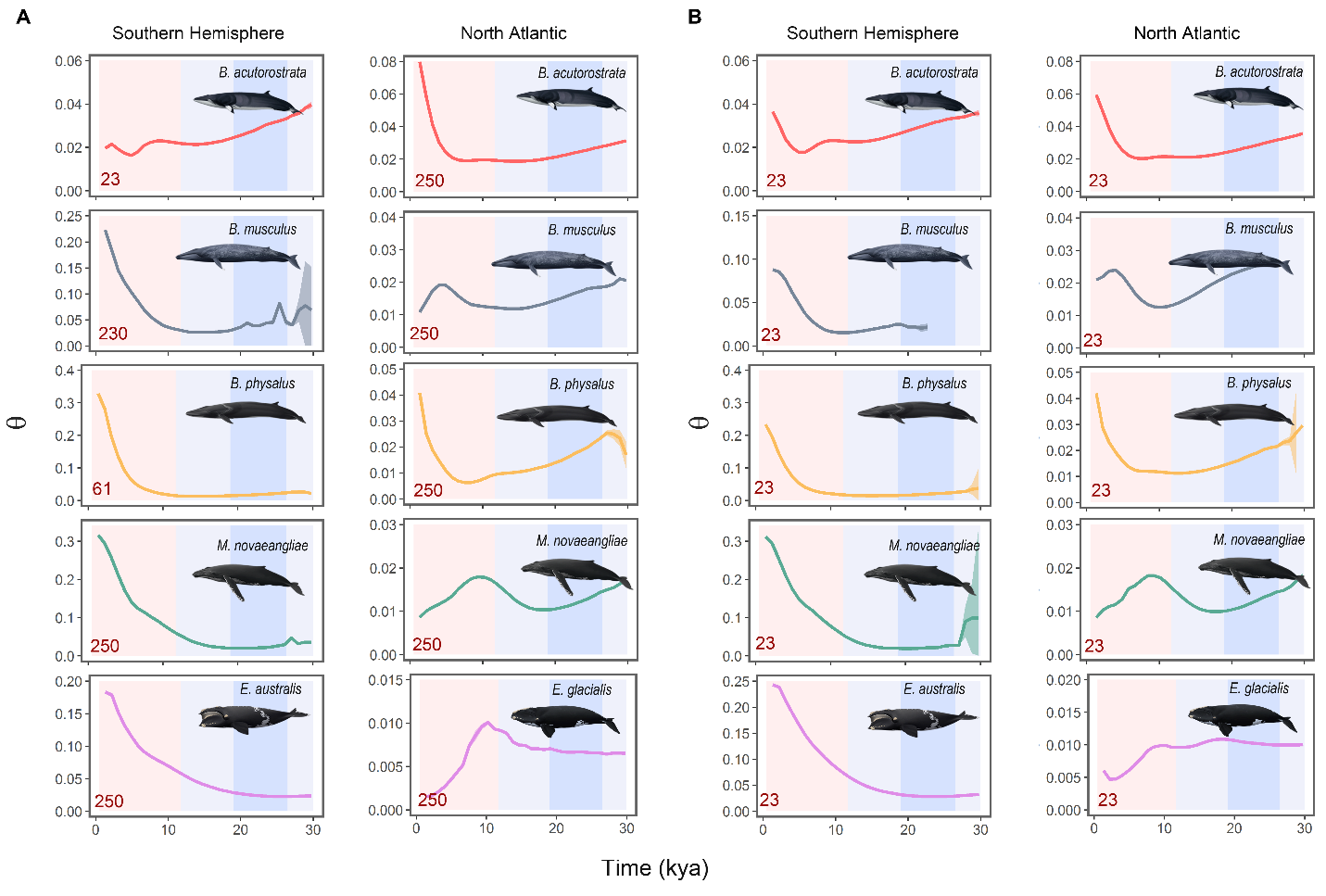


Figure S1. Changes in $\boldsymbol{\Theta}$estimated from two different values of random sub-sampling of mitochondrial DNA sequences. Temporal change in $\boldsymbol{\Theta}$estimated in baleen whales from a maximum random sub-sampling at: (A) 250 mitochondrial DNA sequences and (B) 23 mitochondrial DNA sequences. Values on the X-axis denote the time in thousands of years ago (kya) and the Y-axis the estimate of$\boldsymbol{\Theta}$. The red number at the bottom-right of each plot denotes the sample size employed for each estimate. Note the different scales of the values on the vertical axis in genetic diversity ($\boldsymbol{\Theta}$), and the uneven sample size in some populations. Red and blue shading denotes the Holocene and Pleistocene, respectively. The darkest blue indicates the LGM.


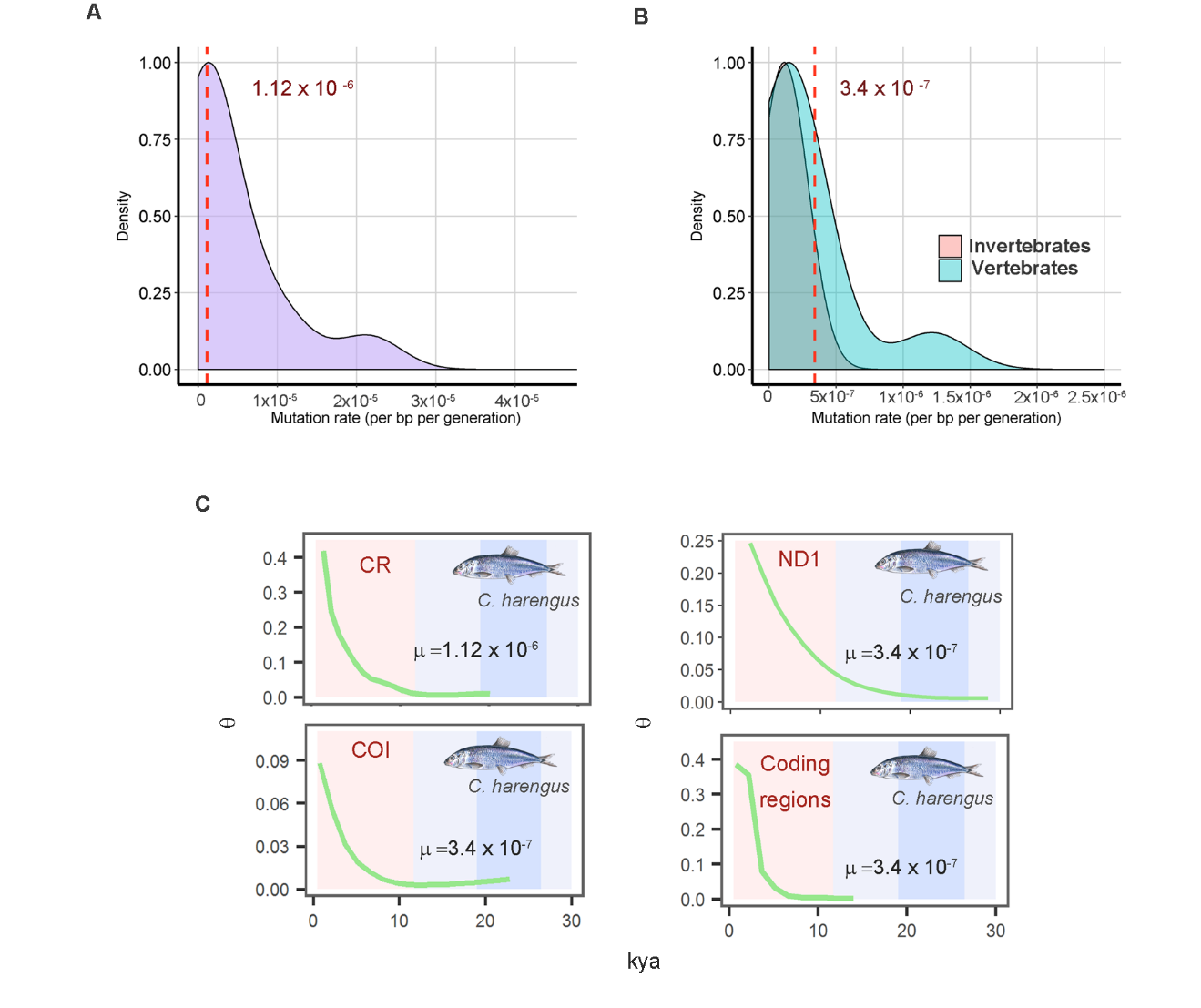


Figure S2. Distribution of reported generational mutation rate estimates for coding and non-coding mtDNA and comparison of the changes in $\boldsymbol{\Theta}$. Distribution of reported generational mutation rate estimates for (A) mtDNA control region (non-coding) and (B) coding mtDNA (including estimates for the entire genome) for vertebrates and invertebrates. Distributions based upon the mutation rates listed in Table S1.1-2. The Y-axis denotes the probability of the X-values based on a kernel density estimation. The red vertical line and inserted rate denotes the chosen mutation rates. (C) Estimated demographic history employing different mitochondrial genes in North Atlantic herring (*Clupea harengus*). Values on the horizontal axis denote the time in thousands of years ago (kya). Vertical axis: estimates of$\boldsymbol{\Theta}$. Note the different scales of the vertical axis. CR: mtDNA control region, COI: mtDNA cytochrome *c* oxidase, subunit 1, ND1: mtDNA NADH dehydrogenase, subunit 1. Coding regions: entire mitochondrial genome excluding the control region. *µ*: the generational mutation rate applied in the analysis. The red- and light blue-shaded areas represent the Holocene and Pleistocene period, respectively. The dark blue-shaded area indicates the LGM.


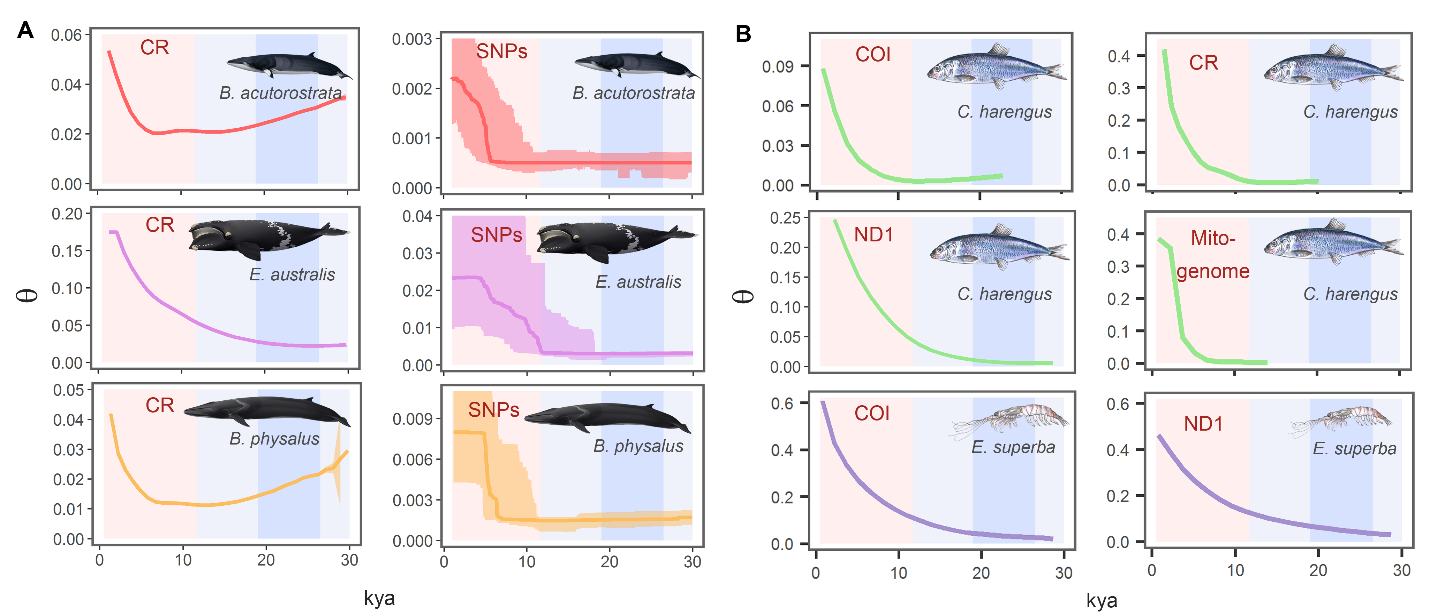
Figure S3. Changes in $\boldsymbol{\Theta}$ estimated from different mitochondrial genes and genome-wide SNP genotypes. (A) Temporal change in $\boldsymbol{\Theta}$ based upon mitochondrial and nuclear DNA data in the common minke whale, the southern right whale and the fin whale. (B) Estimated change in $\boldsymbol{\Theta}$ based upon data from different mitochondrial genes in North Atlantic herring and Antarctic krill. CR: mtDNA control region, mtDNA COI: cytochrome *c* oxidase subunit 1, mtDNA ND1: NADH dehydrogenase subunit 1. Mitogenome: entire mitochondrial genome. SNPs: genome-wide single nucleotide polymorphism genotypes at a minimum coverage of x2. The red- and light blue-shaded areas represent the Holocene and Pleistocene period, respectively. The dark blue-shaded area indicates the LGM. Kya: thousands of years ago. Note the different scales on the vertical axis.


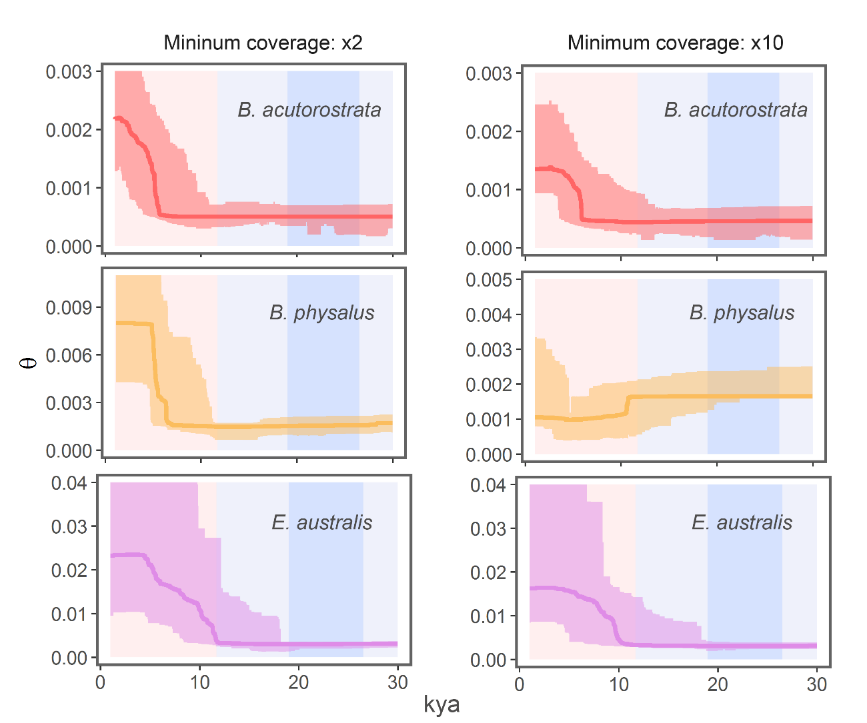


Figure S4. Changes in $\boldsymbol{\Theta}$ estimated from genome-wide SNP genotypes of two different levels of minimum coverage. Estimated demographic history employing genome-wide SNP genotypes with a minimum coverage at x2 and x10 in the North Atlantic common minke whale (top), the North Atlantic fin whale (middle) and the southern right whale (bottom). Values on the X-axis denote the time in thousands of years ago (kya) and the Y-axis the estimate of$\boldsymbol{\Theta}$. Red and blue shading denotes the Holocene and Pleistocene, respectively. The darkest blue indicates the LGM.


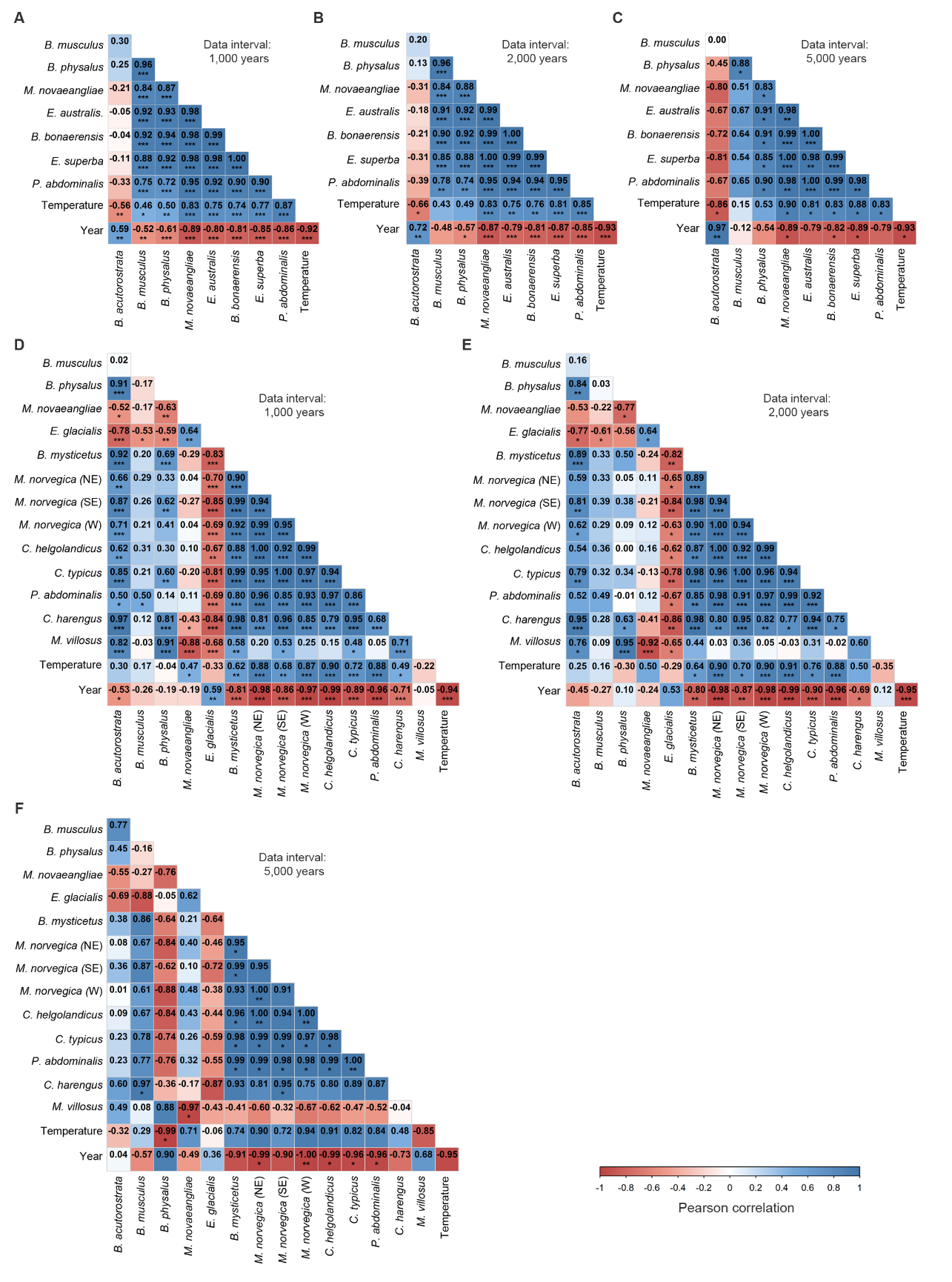


Figure S5. Pairwise Pearson’s correlations among baleen whale and prey estimates of$\boldsymbol{\Theta}$, temperature changes and time. (A - C) Southern Hemisphere, (D - F) North Atlantic Ocean. Blue: positive correlation, red: negative correlation. *, **, *** denotes p-values below 0.05, 0.005, 0.0005, respectively. Time interval between observations was 1,000, 2,000 or 5,000 years.

**
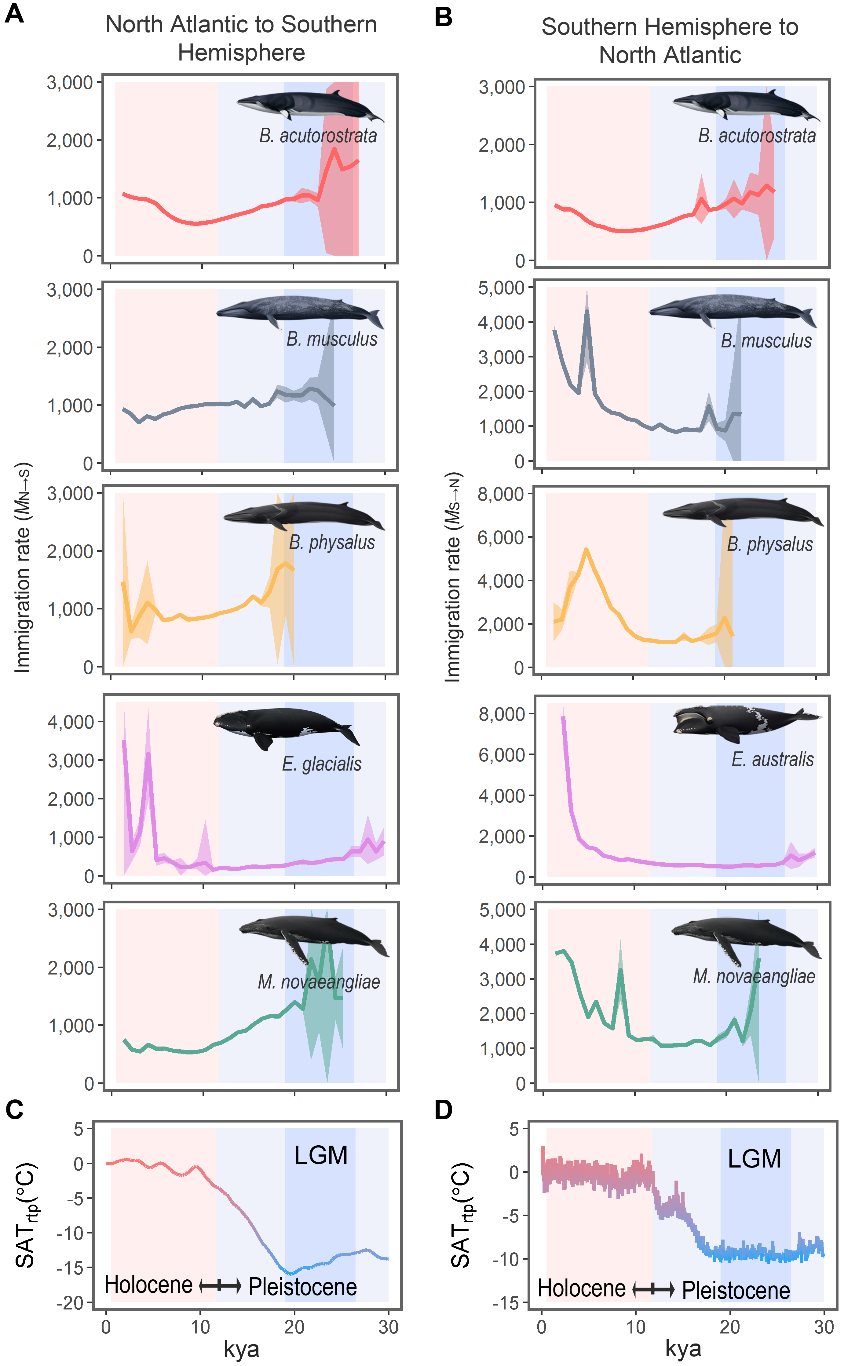
**

Figure S6. Estimates of changes in *M* between North Atlantic Ocean and Southern Hemisphere con-specific baleen whale populations. (A) Immigration rate from North to South ($\boldsymbol{M}_{\boldsymbol{N>S}}$) and (B) immigration rate from South to North ($\boldsymbol{M}_{\boldsymbol{S>N}}$). Note different scales for$\boldsymbol{M}$. (C - D) Historical surface air temperature relative to present temperature (SAT_RTP_) in degrees Celsius (°C) for the Northern (C) and Southern Hemisphere (D). Kya: thousands of years ago. Red and blue shading denotes the Holocene and Pleistocene, respectively. The darkest blue indicates the LGM.

Table S1.

**Prior distributions of estimation parameters in MIGRATE-N.**

| **Species** | **Marker** | **ti:tv** | **Parameter** $\Theta$ | | | | **Parameter *M*** | | | |
| --- | --- | --- | --- | --- | --- | --- | --- | --- | --- | --- |
|  |  |  | **Priors** | | **Starting parameters** | | **Priors** | | **Starting parameters** | |
|  |  |  | **Maximum** | **Delta** | ***Θ* _S_** | ***Θ* _N_** | **Maximum** | **Delta** | ***M*_N>S_** | **M_S>N_** |
| *B. acutorostrata* | CR | 7.7 | 0.1 | 0.01 | 0.01 | 0.03 | 200 | 20 | 50 | 1 |
| *B. musculus* | CR | 19.7 | 0.1 | 0.01 | 0.06 | 0.01 | 300 | 30 | 95 | 1 |
| *B. physalus* | CR | 19.8 | 0.2 | 0.02 | 0.09 | 0.02 | 100 | 10 | 0 | 20 |
| *M. novaeangliae* | CR | 25.4 | 0.15 | 0.015 | 0.07 | 0.007 | 250 | 25 | 60 | 20 |
| *E. glacialis* | CR | 119.5 | 0.1 | 0.01 | 0.04 | 0.003 | 300 | 30 | 1 | 25 |
| *B. mysticetus* | CR | 9.6 | 0.1 | 0.01 |  | 0.035 |  |  |  |  |
| *B. borealis* | CR | 25.2 | 0.4 | 0.04 | 0.22 |  |  |  |  |  |
| *E. superba* | COI | 8.2 | 0.5 | 0.05 | 0.19 |  |  |  |  |  |
| *M. norvegica* | ND1 | 5 | 0.1 | 0.01 |  | 0.02 (NE-NA) 0.017 (SE-NA) 0.012 (W-NA) |  |  |  |  |
| *C. helgolandicus* | 16S | 6.5 | 0.1 | 0.01 |  | 0.02 |  |  |  |  |
| *C. typicus* | COI | 39.2 | 0.5 | 0.05 |  | 0.2 |  |  |  |  |
| *P. abdominalis* | COI | 6.8 | 0.2 | 0.02 | 0.12 | 0.12 | 500 | 50 | 83 | 83 |
| *C. harengus* | COI | 12.3 | 0.2 | 0.02 |  | 0.036 |  |  |  |  |
| *M. villosus* | CYTB | 7.4 | 0.05 | 0.005 |  | 0.02 |  |  |  |  |
| *C. harengus* | CR | 5.6 | 0.4 | 0.04 |  | 0.16 |  |  |  |  |
| *C. harengus* | ND1 | 12.3 | 0.2 | 0.02 |  | 0.04 |  |  |  |  |
| *C. harengus* | Mitogenome | 11.3 | 0.2 | 0.02 |  | 0.08 |  |  |  |  |
| *E. superba* | ND1 | 7.7 | 0.3 | 0.03 | 0.15 |  |  |  |  |  |

Notes: CR: control region, COI: cytochrome *c* oxidase, subunit 1, ND1: NADH dehydrogenase, subunit 1, CYTB: cytochrome *b*, 16S: 16S rDNA of the mtDNA and mitogenome: entire mitochondrial genome excluding the control region. The transition:transversion rate (ti:tv). The prior parameters and the starting parameter values are shown for $\Theta$ and$M$. A uniform distribution and a minimum prior of zero were employed for all priors. $\Theta_{N}$: $\Theta$ North Atlantic population, $\Theta_{S}$: $\Theta$ Southern Hemisphere population, $M_{N>S}$: immigration rate from the North Atlantic Ocean into the Southern Hemisphere and $M_{S>N}$*:* immigration rate from the Southern Hemisphere into North Atlantic Ocean. NE-NA: northeastern North Atlantic, SE-NA: southeastern North Atlantic, W-NA western North Atlantic.

Table S2.

**Summary of mutation rates estimated for the mitochondrial DNA control region sequence.**

| **Marker** | **Species** | **Mutation rate**  **bp/year**  **(95 % CI)** | **Reference** | **Generation time** | **Mutation rate bp/generation**  **(95 % CI)** |
| --- | --- | --- | --- | --- | --- |
| CR | *Eschrichtius robustus* | 5.40x10^-8^  (4.96x10^-8^ - 6.16x10^-8^) | (Alter & Palumbi, 2009) | 25.76 | **1.39x10^-6^**  (1.28x10^-6^ - 1.59x10^-6^) |
| CR | *Eschrichtius robustus* | 4.80x10-^8^  (4.32x10^-8^ - 5.36x10^-8^) | (Alter & Palumbi, 2009) | 25.76 | **1.24x10^-6^**  (1.11x10^-6^ - 1.38x10^-6^) |
| CR | *Balaenoptera acutorostrata* | 5.00x10^-08^  (4.70x10^-8^ - 5.39x10^-8^) | (Alter & Palumbi, 2009) | 21.22 | **1.06x10^-6^**  (9.97x10^-7^ - 1.14x10^-6^) |
| CR | *Balaenoptera acutorostrata* | 5.30x10^-8^  (4.94x10^-8^ - 5.66x10^-8^) | (Alter & Palumbi, 2009) | 21.22 | **1.12x10^-6^**  (1.05x10^-6^ - 1.20x10^-6^) |
| CR | *Balaenoptera acutorostrata* | 4.40x10^-8^  (4.00x10^-8^ - 4.69x10^-8^) | (Alter & Palumbi, 2009) | 21.22 | **9.34x10^-7^**  (8.49x10^-7^ - 9.95x10^-7^) |
| CR | *Megaptera novaeangliae* | 4.60x10^-8^  (3.59x10^-8^ - 5.50x10^-8^) | (Alter & Palumbi, 2009) | 26.87 | **1.24x10^-6^**  (9.65x10^-7^ - 1.48x10^-6^) |
| CR | *Megaptera novaeangliae* | 5.20x10^-8^  (4.12x10^-8^ - 6.32x10^-8^) | (Alter & Palumbi, 2009) | 26.87 | **1.40x10^-6^**  (1.11x10^-6^ - 1.70x10^-6^) |
| CR | *Megaptera novaeangliae* | 8.50x10^-9^  (7.0x10^-9^ – 1.0x10^-8^) | (Baker et al., 1993) | 26.87 | **2.28x10^-7^**  (1.88x10^-7^ - 2.69x10^-7^) |
| CR | *Balaena mysticetus* | 1.50x10^-7^  (n/a) | (Ho et al., 2007) | 52.67 | **7.90x10^-6^**  (n/a) |
| CR | *Balaena mysticetus* | 2.10x10^-7^  (1.22x10^-7^ - 3.03x10^-7^) | (Ho et al., 2007) | 52.67 | **1.11x10^-5^**  (6.43x10^-6^ - 1.60x10^-5^) |
| CR | *Balaena mysticetus* | 2.00x10^-8^  (1.20x10^-8^ - 3.70x10^-8^) | (Rooney et al., 2001) | 52.67 | **1.05x10^-6^**  (6.32x10^-7^ - 1.95x10^-6^) |
| CR | *Balaena mysticetus* | 4.11x10^-7^  (2.07x10^-7^ - 6.49x10^-7^) | (Foote et al., 2013) | 52.67 | **2.16x10^-5^**  (1.09x10^-5^ - 3.42x10^-5^) |

Notes: Mutation rates per base pair per year (bp/year) per generation (bp/generation). CR: mtDNA control region. Numbers in parentheses denote the 95% confidence interval of the mutation rate estimates. Generation time represents the average estimates reported by Pacifici *et al*. (2013) and Taylor *et al.* (Taylor et al., 2007). n/a: not available.

Table S3.

**Summary of mutation rates estimated for the coding genes of the mitochondrial DNA and for the entire mitochondrial genome.**

| **Marker** | **Species** | **Mutation rate**  **bp/year**  **(95 % CI)** | **Reference** | **Generation time** | **Mutation rate bp/generation**  **(95 % CI)** | **Reference** |
| --- | --- | --- | --- | --- | --- | --- |
| **Vertebrates** |  |  |  |  |  |  |
| CYTB | *Eschrichtius robustus* | 4.00x10^-9^  (3.87x10^-9^ - 4.13x10^-9^) | (Alter & Palumbi, 2009) | 25.76 (Pacifici et al., 2013; Taylor et al., 2007) | **1.03x10^-7^**  (9.97x10^-8^ - 1.06x10^-7^) |  |
| CYTB | *Balaenoptera borealis* | 7.00x10^-9^  (3.00x10^-9^ - 1.20x10^-8^) | (Nabholz et al., 2008) | 22.05 (Pacifici et al., 2013; Taylor et al., 2007) | **1.54x10^-7^**  (6.62x10^-8^ - 2.65x10^-7^) |  |
| CYTB | *Megaptera novaeangliae* | 8.00x10^-9^  (5.00x10^-9^ - 2.00x10^-8^) | (Nabholz et al., 2008) | 26.87 (Pacifici et al., 2013; Taylor et al., 2007) | **2.15x10^-7^**  (1.34x10^-7^ - 5.37x10^-7^) |  |
| Mitogenome | *Ursus maritimus* | 1.12x10^-8^  (8.96x10^-9^ - 1.50x10^-8^) | (Krause et al., 2008) | 15 (Pacifici et al., 2013) | **1.68x10^-7^**  (1.34x10^-7^ - 2.25x10^-7^) |  |
| Mitogenome | *Orcinus orca* | 2.60x10^-9^  (1.50x10^-9^ - 3.83x10^-9^) | (Morin et al., 2010) | 31.8 (Pacifici et al., 2013; Taylor et al., 2007) | **8.27x10^-8^**  (4.77x10^-8^ - 1.22x10^-7^) |  |
| Coding mtDNA | *Homo sapiens* | 1.70x10^-8^  (n/a) | (Ingman et al., 2000) | 20 (Nachman & Crowell, 2000) | **3.40x10^-7^**  (n/a) |  |
| Coding mtDNA | *Homo sapiens* | 1.26x10^-8^  (1.18x10^-08^ - 1.34x10^-8^) | (Mishmar et al., 2003) | 20 (Nachman & Crowell, 2000) | **2.52x10^-7^**  (2.36x10^-7^ - 2.68x10^-7^) |  |
| Coding mtDNA | *Homo sapiens* | 6.09 x10^-8^  (n/a) | (Howell et al., 2003) | 20 (Nachman & Crowell, 2000) | **1.22 x10^-6^**  (n/a) |  |
| CYTB | *Anisotremus* spp*.* |  |  |  | **1.60x10^-8^**  (1.50x10^-8^ - 1.70x10^-8^) | (Bernardi & Lape, 2005) |
| Mitogenome | *Clupea harengus* | 4.70x10^-9^  (3.50x10^-9^ - 6.00x10^-9^) | (Teacher et al., 2012) | 6.5 (Lorance et al., 2015) | **3.06x10^-8^**  (2.28x10^-8^ - 3.90x10^-8^) |  |
| **Invertebrates** |  |  |  |  |  |  |
| COI | *Melarhaphe neritoides* | 5.82x10^-5^ | (Fourdrilis et al., 2016) | 3.42 (Fourdrilis et al., 2016) | **1.99x10^-4^** | (Fourdrilis et al., 2016) |
| COI | *Mytilus edulis* |  |  |  | **9.51x10^-8^**  (5.67x10^-8^ – 1.34x10^-7^) | (Wares & Cunningham, 2001) |
| COI | *Asteria rubens* |  |  |  | **4.84 x10^-8^**  (3.12x10^-8^ – 6.56x10^-8^) | (Wares & Cunningham, 2001) |
| COI | *Nucella lapillus* |  |  |  | **4.43 x10^-8^**  (2.33x10^-8^ – 6.53x10^-8^) | (Wares & Cunningham, 2001) |
| COI | *Littorina obtusata* |  |  |  | **2.49 x10^-8^**  (0.00 – 5.04x10^-8^) | (Wares & Cunningham, 2001) |
| COI | *Semibalanus balanoides* |  |  |  | **2.76 x10^-8^**  (1.52x10^-8^ – 4.00x10^-8^) | (Wares & Cunningham, 2001) |
| CYTB | *Paralithodes camtschaticus* | 5.00x10^-9^  (n/a) | (Grant & Cheng, 2012) | 5 (Grant & Cheng, 2012) | **2.50x10^-8^**  (n/a) |  |
| Mitogenome | *Daphnia pulex* |  |  |  | **1.73x10^-7^**  (n/a) | (Xu et al., 2012) |
| Mitogenome | *Daphnia pulex* |  |  |  | **1.37x10^-7^**  (n/a) | (Xu et al., 2012) |
| Mitogenome | *Caenorhabditis elegans* |  |  |  | **1.60x10^-7^**  (1.29x10^-7^ - 1.91x10^-7^) | (Denver et al., 2000) |
| Mitogenome | *Drosophila melanogaster* |  |  |  | **6.20x10^-8^**  (3.00x10^-8^ - 1.14x10-^7^) | (Haag-Liautard et al., 2008) |

Notes: Estimates are in mutations per base pair per year (bp/year) and per generation (bp/generation). COI: cytochrome c oxidase, subunit I, CYTB: cytochrome *b* of the mtDNA, Coding mtDNA: all coding DNA sequences in the mtDNA genome (i.e., excluding non-coding region). Mitogenome: entire mtDNA genome. Numbers in parentheses denote the 95% confidence interval of the estimated mutation rate. Reference of the mutation rate per bp/year or bp/generation. Generation time: average generation length estimate. n/a: not available.

Table S4.

**Species and sample sizes.**

| Species | Common name | Sampling region | Marker | n | Sequence length/Number of SNPs | Source |
| --- | --- | --- | --- | --- | --- | --- |
| *C. harengus* | Atlantic herring | ENA | COI | 98 | 1,551 | (Teacher et al., 2012) |
| *C. harengus* | Atlantic herring | ENA | CR | 98 | 1,055 | (Teacher et al., 2012) |
| *C. harengus* | Atlantic herring | ENA | ND1 | 98 | 975 | (Teacher et al., 2012) |
| *C. harengus* | Atlantic herring | ENA | Mitogenome | 98 | 15,653 | (Teacher et al., 2012) |
| *E. superba* | Antarctic krill | SO | COI | 640 | 593 | (Deagle et al., 2015; Goodall-Copestake et al., 2010) |
| *E. superba* | Antarctic krill | SO | NDI | 139 | 494 | (Deagle et al., 2015) |
| *B. acutorostrata* | Common minke whale | NA | CR | 867 | 322 | This study |
| *B. acutorostrata* | Common minke whale | NA | SNPs | 27 | 14,304 (x10)  24,988 (x2) | This study |
| *E. australis* | Southern right whale | SA | CR | 481 | 381 | This study, (Valenzuela et al., 2009) |
| *E. australis* | Southern right whale | ESA | SNPs | 45 | 31,482 (x10)  68,575 (x2) | This study |
| *B. physalus* | Fin whale | WNA | CR | 280 | 391 | This study |
| *B. physalus* | Fin whale | NA | SNPs | 28 | 29,544 (x10)  56,325 (x2) | This study |

Notes: Species, sampling region, molecular marker, sample size (n), sequence length in number of bp or number of estimated SNPs (i.e., number of inferred polymorphic sites from the site frequency spectrum) for each species with minimum coverage at x10 and x2. CR: control region, COI: the cytochrome *c* oxidase subunit 1, ND1: the NADH dehydrogenase subunit 1 of the mtDNA, Mitogenome: entire mitochondrial genome excluding the control region, SNPs: single nucleotide polymorphism genotypes. NA: North Atlantic, ENA: eastern North Atlantic, WNA: western North Atlantic, SA: South Atlantic, SO: Southern Ocean and ESA: eastern South Atlantic.

Table S5.

**Genetic diversity indices of the baleen whales and prey species for the North Atlantic and Southern Ocean.**

| **Species** | **Region** | **n** | **Marker** | **bp** | **GM** | **S** | **h** | **H** ±**SD** | **π** ± **SD** | **Tajima’s D** | **Fu's F** |
| --- | --- | --- | --- | --- | --- | --- | --- | --- | --- | --- | --- |
| **North Atlantic** |  |  |  |  |  |  |  |  |  |  |  |
| **Baleen whales** |  |  |  |  |  |  |  |  |  |  |  |
| *B. acutorostrata* | NA | 931 | CR | 322 | 0 | 27 | 49 | 0.901 ± 0.00004 | 0.0076 ± nd | -0.933 | -30.83 |
| *B. musculus* | NA | 325 | CR | 404 | 1 | 18 | 19 | 0.887 ± 0.008 | 0.0085 ± 0.00036 | 0.526 | -1.13 |
| *B. physalus* | WNA | 280 | CR | 391 | 0 | 30 | 31 | 0.882 ± 0.011 | 0.0101 ± 0.00049 | -0.582 | -7.82 |
| *M. novaeangliae* | WI | 1086 | CR | 396 | 4 | 25 | 24 | 0.855 ± 0.006 | 0.0168 ± nd | 2.386* | 4.10 |
| *E. glacialis* | WNA | 269 | CR | 381 | 0 | 7 | 5 | 0.698 ± 0.016 | 0.0052 ± 0.00018 | 1.536 | 4.55 |
| *B. mysticetus* | WNA | 395 | CR | 454 | 1 | 43 | 50 | 0.924 ± 0.007 | 0.012 ± 0.00052 | -0.623 | -16.85 |
| **Prey species** |  |  |  |  |  |  |  |  |  |  |  |
| *M. norvegica* | NA | 834* | ND1 | 155 | 0 | 22 | 31 | 0.595 ± 0.009 | 0.0054 ± nd | -1.779* | -33.56 |
| *C. helgolandicus* | ENA | 218 | 16S | 408 | 0 | 26 | 27 | 0.55 ± 0.038 | 0.0032 ± 0.00031 | -1.971* | -23.61 |
| *C. typicus* | NA | 79 | COI | 560 | 0 | 105 | 70 | 0.99 ± 0.007 | 0.0105 ± 0.00078 | -2.488* | -103.27 |
| *P. abdominalis* | NA | 130 | COI | 441 | 0 | 135 | 81 | 0.981 ± 0.006 | 0.0816 ± 0.00636 | 0.429 | -13.98 |
| *C. harengus* | ENA | 98 | COI | 1,551 | 0 | 97 | 67 | 0.979 ± 0.007 | 0.0036 ± 0.0002 | -2.315* | -34.25 |
| *M. villosus* | WNA | 41 | CYTB | 572 | 0 | 32 | 22 | 0.943 ± 0.02 | 0.0135 ± 0.0024 | 0.114 | -4.83 |
|  |  |  |  |  |  |  |  |  |  |  |  |
| **Southern Hemisphere** |  |  |  |  |  |  |  |  |  |  |  |
| **Baleen whales** |  |  |  |  |  |  |  |  |  |  |  |
| *B. acutorostrata* | WSA, SO | 23 | CR | 322 | 0 | 18 | 9 | 0.9117 ± 0.025 | 0.0192 ± 0.00221 | 0.968 | 1.12 |
| *B. musculus* | SO | 230 | CR | 404 | 74 | 45 | 51 | 0.967 ± 0.003 | 0.0173 ± 0.00057 | -0.785 | -22.82 |
| *B. physalus* | SO | 61 | CR | 391 | 0 | 34 | 47 | 0.991 ± 0.005 | 0.013 ± 0.00072 | -0.984 | -33.65 |
| *M. novaeangliae* | SA | 500 | CR | 396 | 10 | 67 | 92 | 0.973 ± 0.002 | 0.0235 ± 0.00029 | -0.373 | -42.83 |
| *E. australis* | SA | 481 | CR | 381 | 2 | 50 | 51 | 0.961 ± 0.002 | 0.0246 ± 0.00027 | 0.718 | -4.76 |
| *B. bonaerensis* | WSA, SO | 180 | CR | 337 | 3 | 59 | 114 | 0.986 ± 0.00002 | 0.0153 ± 0.00072 | -1.674 | -183.15 |
| **Prey species** |  |  |  |  |  |  |  |  |  |  |  |
| *E. superba* | SO | 640 | COI | 593 | 0 | 140 | 381 | 0.979 ± 0.003 | 0.0109 ± nd | -2.160* | -31.67 |
| *P. abdominalis* | SA, WIO | 231 | COI | 441 | 0 | 150 | 98 | 0.949 ± 0.01 | 0.1187 ± 0.00193 | 1.703 | -2.20 |

Notes: table includes the sampling region, the number of sequences per data set (n), the molecular marker, the length of the sequence in number of base pairs (bp), the number of gaps and missing data (GM), the number of segregating (polymorphic) sites (S), the number of haplotypes (h) and the haplotype diversity (H) and the nucleotide diversity (π) with their standard deviation (SD), as well as the Tajima’s D and Fu’s F statistic.

**References**

Alter, S. E., & Palumbi, S. R. (2009). Comparing evolutionary patterns and variability in the mitochondrial control region and cytochrome *b* in three species of baleen whales. *Journal of Molecular Evolution, 68*, 97–111.

Aquadro, C. F., & Greenberg, B. D. (1983). Human mitochondrial DNA variation and evolution: Analysis of nucleotide sequences from seven individuals. *Genetics, 103*(2), 287. Retrieved from <http://www.genetics.org/content/103/2/287.abstract>

Archer, F. I., Morin, P. A., Hancock-Hanser, B. L., Robertson, K. M., Leslie, M. S., Bérubé, M., . . . Taylor, B. L. (2013). Mitogenomic phylogenetics of fin whales (*Balaenoptera physalus spp.*): Genetic evidence for revision of subspecies. *PLoS ONE, 8*(5), e63396. doi:10.1371/journal.pone.0063396

Bacon, C. D., Silvestro, D., Jaramillo, C., Smith, B. T., Chakrabarty, P., & Antonelli, A. (2015). Biological evidence supports an early and complex emergence of the Isthmus of Panama. *Proceedings of the National Academy of Sciences of the United States of America, 112*(19), 6110-6115. doi:10.1073/pnas.1423853112

Baker, C. S., Perry, A., Bannister, J. L., Weinrich, M. T., Abernethy, R. B. M. C. J., Lien, J., . . . Palumbi, S. P. (1993). Abundant mitochondrial DNA variation and world-wide population structure in humpback whales. *Proceedings of the National Academy of Sciences of the United States of America, 90*, 8239-8243.

Beerli, P. (2009). How to use MIGRATE or why are Markov chain Monte Carlo programs difficult to use? In G. Bertorelle, M. W. Bruford, H. C. Hauffe, A. Rizzoli, & C. Vernesi (Eds.), *Population genetics for animal conservation* (pp. 39-77). United Kingdom: Cambridge University Press.

Bernardi, G., & Lape, J. (2005). Tempo and mode of speciation in the Baja California disjunct fish species *Anisotremus davidsonii*. *Molecular Ecology, 14*(13), 4085-4096. doi:10.1111/j.1365-294X.2005.02729.x

Castellani, C., Lindley, A. J., Wootton, M., Lee, C. M., & Kirby, R. R. (2012). Morphological and genetic variation in the North Atlantic copepod, *Centropages typicus*. *Journal of the Marine Biological Association of the United Kingdom, 92*(1), 99-106. doi:10.1017/S0025315411000932

Colbeck, G. J., Turgeon, J., Sirois, P., & Dodson, J. J. (2011). Historical introgression and the role of selective vs. neutral processes in structuring nuclear genetic variation (AFLP) in a circumpolar marine fish, the capelin (*Mallotus villosus*). *Molecular Ecology, 20*(9), 1976-1987. doi:10.1111/j.1365-294X.2011.05069.x

Deagle, B. E., Faux, C., Kawaguchi, S., Meyer, B., & Jarman, S. N. (2015). Antarctic krill population genomics: apparent panmixia, but genome complexity and large population size muddy the water. *Molecular Ecology, 24*(19), 4943-4959. doi:10.1111/mec.13370

Denver, D. R., Morris, K., Lynch, M., Vassilieva, L. L., & Thomas, W. K. (2000). High direct estimate of the mutation rate in the mitochondrial genome of *Caenorhabditis elegans*. *Science, 289*(5488), 2342-2344. doi:10.1126/science.289.5488.2342

Felsenstein, J. (2006). Accuracy of coalescent likelihood estimates: Do we need more sites, more sequences, or more loci? *Molecular Biology and Evolution, 23*(3), 691-700. doi:DOI 10.1093/molbev/msj079

Foote, A. D., Kaschner, K., Schultze, S. E., Garilao, C., Ho, S. Y. W., Post, K., . . . Gilbert, M. T. P. (2013). Ancient DNA reveals that bowhead whale lineages survived Late Pleistocene climate change and habitat shifts. *Nature Communications, 4*, 1677. doi:10.1038/ncomms2714

Fourdrilis, S., Mardulyn, P., Hardy, O. J., Jordaens, K., Martins, A. M. D., & Backeljau, T. (2016). Mitochondrial DNA hyperdiversity and its potential causes in the marine periwinkle *Melarhaphe neritoides* (Mollusca: Gastropoda). *Peerj, 4*. doi:10.7717/peerj.2549

Goodall-Copestake, W. P., Perez-Espona, S., Clark, M. S., Murphy, E. J., Seear, P. J., & Tarling, G. A. (2010). Swarms of diversity at the gene cox1 in Antarctic krill. *Heredity, 104*(5), 513-518. doi:10.1038/hdy.2009.188

Grant, W. S., & Cheng, W. (2012). Incorporating deep and shallow components of genetic structure into the management of Alaskan red king crab. *Evolutionary Applications, 5*(8), 820-837. doi:10.1111/j.1752-4571.2012.00260.x

Haag-Liautard, C., Coffey, N., Houle, D., Lynch, M., Charlesworth, B., & Keightley, P. D. (2008). Direct estimation of the mitochondrial DNA mutation rate in *Drosophila melanogaster*. *PLoS Biology, 6*(8), 1706-1714. doi:10.1371/journal.pbio.0060204

Hirai, J., Tsuda, A., & Goetze, E. (2015). Extensive genetic diversity and endemism across the global range of the oceanic copepod *Pleuromamma abdominalis*. *Progress in Oceanography, 138*, 77-90. doi:10.1016/j.pocean.2015.09.002

Ho, S. Y. W., Kolokotronis, S.-O., & Allaby, R. G. (2007). Elevated substitution rates estimated from ancient DNA sequences. *Biology Letters, 3*(6), 702-705. Retrieved from <http://rsbl.royalsocietypublishing.org/content/3/6/702.abstract>

Ho, S. Y. W., & Larson, G. (2006). Molecular clocks: when times are a-changin'. *Trends in Genetics, 22*(2), 79-83. doi:10.1016/j.tig.2005.11.006

Ho, S. Y. W., Phillips, M. J., Cooper, A., & Drummond, A. J. (2005). Time dependency of molecular rate estimates and systematic overestimation of recent divergence times. *Molecular Biology and Evolution, 22*(7), 1561-1568. doi:10.1093/molbev/msi145

Hodgkinson, A., & Eyre-Walker, A. (2011). Variation in the mutation rate across mammalian genomes. *Nature Reviews Genetics, 12*(11), 756-766. doi:10.1038/nrg3098

Howell, N., Smejkal, C. B., Mackey, D. A., Chinnery, P. F., Turnbull, D. M., & Herrnstadt, C. (2003). The pedigree rate of sequence divergence in the human mitochondrial genome: There is a difference between phylogenetic and pedigree rates. *American Journal of Human Genetics, 72*(3), 659-670. doi:10.1086/368264

Ingman, M., Kaessmann, H., Paabo, S., & Gyllensten, U. (2000). Mitochondrial genome variation and the origin of modern humans. *Nature, 408*(6813), 708-713. Retrieved from <Go to ISI>://WOS:000165815200046

Jackson, J. A., Steel, D. J., Beerli, P., Congdon, B. C., Olavarría, C., Leslie, M. S., . . . Baker, C. S. (2014). Global diversity and oceanic divergence of humpback whales (*Megaptera novaeangliae*). *Proceedings of the Royal Society of London, Series B: Biological Sciences, 281*(1786). doi:10.1098/rspb.2013.3222

Krause, J., Unger, T., Nocon, A., Malaspinas, A.-S., Kolokotronis, S.-O., Stiller, M., . . . Hofreiter, M. (2008). Mitochondrial genomes reveal an explosive radiation of extinct and extant bears near the Miocene-Pliocene boundary. *BMC Evolutionary Biology, 8*(1), 220. doi:10.1186/1471-2148-8-220

LeDuc, R. G., Martien, K. K., Morin, P. A., Hedrick, N., Robertson, K. M., Taylor, B. L., . . . George, J. C. (2008). Mitochondrial genetic variation in bowhead whales in the western Arctic. *Journal of Cetacean Research and Management, 10*(2), 93-97.

Lessios, H. A. (2008). The Great American Schism: Divergence of marine organisms after the rise of the Central American Isthmus. *Annual Review of Ecology Evolution and Systematics, 39*, 63-91. doi:10.1146/annurev.ecolsys.38.091206.095815

Liu, X. M., & Fu, Y. X. (2015). Exploring population size changes using SNP frequency spectra. *Nature Genetics, 47*(5), 555-U172. doi:10.1038/ng.3254

Lorance, P., Nedreaas, K., Fernandes, P., Kjellén, N., & Florin, A. (2015). *Clupea harengus*. The IUCN Red List of Threatened Species 2015. *e.T155123A45074983*. Retrieved from <http://www.iucnredlist.org/details/155123/1>

Malik, S., Brown, M. W., Kraus, S. D., Knowlton, A. R., Hamilton, P. K., & White, B. N. (1999). Assessment of mitochondrial DNA structuring and nursery use in the North Atlantic right whale (*Eubalaena glacialis*). *Canadian Journal of Zoology, 77*(8), 1217-1222.

Mishmar, D., Ruiz-Pesini, E., Golik, P., Macaulay, V., Clark, A. G., Hosseini, S., . . . Wallace, D. C. (2003). Natural selection shaped regional mtDNA variation in humans. *Proceedings of the National Academy of Sciences of the United States of America, 100*(1), 171-176. doi:10.1073/pnas.0136972100

Montes, C., Cardona, A., Jaramillo, C., Pardo, A., Silva, J. C., Valencia, V., . . . Nino, H. (2015). Middle Miocene closure of the Central American Seaway. *Science, 348*(6231), 226-229. doi:10.1126/science.aaa2815

Morin, P. A., Archer, F. I., Foote, A. D., Vilstrup, J., Allen, E. E., Wade, P., . . . Harkins, T. (2010). Complete mitochondrial genome phylogeographic analysis of killer whales (*Orcinus orca*) indicates multiple species. *Genome Research, 20*(7), 908-916. Retrieved from <http://genome.cshlp.org/content/20/7/908.abstract>

Nabholz, B., Glemin, S., & Galtier, N. (2008). Strong variations of mitochondrial mutation rate across mammals - the longevity hypothesis. *Molecular Biology and Evolution, 25*(1), 120-130. doi:10.1093/molbev/msm248

Nabholz, B., Glemin, S., & Galtier, N. (2009). The erratic mitochondrial clock: variations of mutation rate, not population size, affect mtDNA diversity across birds and mammals. *Bmc Evolutionary Biology, 9*(1), 120-130. doi:10.1186/1471-2148-9-54

Nachman, M. W., & Crowell, S. L. (2000). Estimate of the mutation rate per nucleotide in humans. *Genetics, 156*(1), 297-304. Retrieved from <Go to ISI>://WOS:000089209800024

Pacifici, M., Santini, L., Di Marco, M., Baisero, D., Francucci, L., Marasini, G. G., . . . Rondinini, C. (2013). Generation length for mammals. *Nature Conservation, 5*, 89-94.

Papetti, C., Zane, L., Bortolotto, E., Bucklin, A., & Patarnello, T. (2005). Genetic differentiation and local temporal stability of population structure in the euphausiid *Meganyctiphanes norvegica*. *Marine Ecology Progress Series, 289*, 225-235. doi:10.3354/meps289225

Pastene, L. A., Goto, M., Kanda, N., Zerbini, A. N., Kerem, D., Watanabe, K., . . . Palsbøll, P. J. (2007). Radiation and speciation of pelagic organisms during periods of global warming: the case of the common minke whale, *Balaenoptera acutorostrata*. *Molecular Ecology, 16*(7), 1481-1495. doi:10.1111/j.1365-294X.2007.03244.x

Pluzhnikov, A., & Donnelly, P. (1996). Optimal sequencing strategies for surveying molecular genetic diversity. *Genetics, 144*(3), 1247-1262. Retrieved from <Go to ISI>://WOS:A1996VP82600034

Rooney, A. P., Honeycutt, R. L., & Derr, J. N. (2001). Historical population size change of bowhead whales inferred from DNA sequence polymorphism data. *Evolution, 55*(8), 1678-1685.

Sremba, A. L., Hancock-Hanser, B., Branch, T. A., LeDuc, R. L., & Baker, C. S. (2012). Circumpolar diversity and geographic differentiation of mtDNA in the critically endangered Antarctic blue whale (*Balaenoptera musculus intermedia*). *PLoS ONE, 7*(3), e32579. doi:10.1371/journal.pone.0032579

Taylor, B. L., Chivers, S. J., Larese, J., & Perrin, W. F. (2007). *Generation length and percent mature estimates for IUCN assessments of cetaceans. Administrative report LJ-07-01*. Retrieved from La Jolla, California:

Teacher, A. G. F., Andre, C., Merila, J., & Wheat, C. W. (2012). Whole mitochondrial genome scan for population structure and selection in the Atlantic herring. *BMC Evolutionary Biology, 12*(248). doi:10.1186/1471-2148-12-248

Valenzuela, L. O., Sironi, M., Rowntree, V. J., & Seger, J. (2009). Isotopic and genetic evidence for culturally inherited site fidelity to feeding grounds in southern right whales (*Eubalaena australis*). *Molecular Ecology, 18*(5), 782-791. doi:10.1111/j.1365-294X.2008.04069.x

Wares, J. P., & Cunningham, C. W. (2001). Phylogeography and historical ecology of the North Atlantic intertidal. *Evolution, 55*(12), 2455-2469. Retrieved from <Go to ISI>://WOS:000173528000008

Xu, S., Schaack, S., Seyfert, A., Choi, E. J., Lynch, M., & Cristescu, M. E. (2012). High mutation rates in the mitochondrial genomes of *Daphnia pulex*. *Molecular Biology and Evolution, 29*(2), 763-769. doi:10.1093/molbev/msr243

Yebra, L., Bonnet, D., Harris, R. P., Lindeque, P. K., & Peijnenburg, K. T. C. A. (2011). Barriers in the pelagic: population structuring of *Calanus helgolandicus* and *C. euxinus* in European waters. *Marine Ecology Progress Series, 428*, 135-149. doi:10.3354/meps09056

Zane, L., Ostellari, L., Maccatrozzo, L., Bargelloni, L., Cuzin-Roudy, J., Buchholz, F., & Patarnello, T. (2000). Genetic differentiation in a pelagic crustacean (*Meganyctiphanes norvegica*: Euphausiacea) from the North East Atlantic and the Mediterranean Sea. *Marine Biology, 136*(2), 191-199. doi:10.1007/s002270050676
